# IDO1 inhibits ferroptosis by regulating FTO-mediated m6A methylation and SLC7A11 mRNA stability during glioblastoma progression

**DOI:** 10.1038/s41420-025-02293-3

**Published:** 2025-01-25

**Authors:** Qianting Tian, Guixue Dan, Xuyan Wang, Jiamei Zhu, Chaochun Chen, Dekun Tang, Ziming Wang, Dan Chen, Shan Lei, Chao Yang, Houmei Wang, Bing Guo, Bangming Jin, Tengxiang Chen, Lei Tang

**Affiliations:** 1 Department of Physiology, School of Basic Medical Sciences, Guizhou Medical University, Guiyang, China; 2 Transformation Engineering Research Center of Chronic Disease Diagnosis and Treatment, Guizhou Medical University, Guiyang, China; 3 Guizhou Provincial Key Laboratory of Pathogenesis and Drug Research on Common Chronic Diseases, Guizhou Medical University, Guiyang, China; 4 Guizhou Institute of Precision Medicine, Affiliated Hospital of Guizhou Medical University, Guiyang, China; 5 State Key Laboratory of Functions and Applications of Medicinal Plants, School of Basic Medical Sciences, Guizhou Provincial Engineering Technology Research Center for Chemical Drug R&D, Guizhou Medical University, Guiyang, China

**Keywords:** CNS cancer, Oncogenesis

## Abstract

Indoleamine 2, 3-dioxygenase 1 (IDO1) has been recognized as an enzyme involved in tryptophan catabolism with immunosuppressive ability. This study determined to investigate the impact of IDO1 on glioblastoma multiforme (GBM) cells. Here, we showed that the expression of IDO1 was markedly increased in patients with glioma and associated with GBM progression. IDO1 overexpression suppressed ferroptotic cell death, reduced ROS and lipid peroxide generation in GBM cells. IDO1 expression increased the SLC7A11 mRNA stability through FTO-dependent m6A methylation. Mechanistically, IDO1 promoted the AhR expression and nuclear translocation, thus facilitating AhR recruitment at the promoter regions of FTO gene and negatively regulating its transcription. These findings demonstrate that IDO1 facilitates GBM progression by inhibiting SLC7A11-dependent ferroptosis through an IDO1-AhR-FTO axis-mediated m6A methylation mechanism.

## Introduction

Glioblastoma multiforme (GBM) is the most aggressive cerebral tumor in the central nervous system. The median survival remains <15 months, presenting poor prognoses and considerable risk in patients with GBM, despite advances in surgical intervention, radiotherapy, and chemotherapy [1–3]. Hence, an in-depth clarification of the underlying molecular mechanisms of GBM pathogenesis and an investigation of potential targets for its therapy is urgently needed.

Indoleamine 2, 3-dioxygenase 1 (IDO1) is a rate-limiting enzyme that converts the essential amino acid tryptophan into downstream kynurenine (Kyn), indicated as a novel mechanism for blocking the proliferation and anti-tumor activity of immune cells by activating the aryl hydrocarbon receptor (AhR) [4, 5]. GBM cells typically do not express IDO1, but it is inducible by interferons secreted by tumor-infiltrating T cells [6]. Compelling evidence indicated that higher GBM-infiltrating T-cell levels were consequently related to increased tumor IDO1 expression and a corresponding decrease in overall survival (OS) for patients with GBM [7]. These observations motivated several investigations of pharmacologic enzyme-inhibitor approaches targeting IDO1 metabolic activity [8, 9]. However, randomized clinical trials assessing this approach in patients with cancer revealed no objective survival benefits to date [5, 10]. Reportedly, Kyn, the major metabolic product of IDO1, and its downstream metabolites of 3-hydroxy-kynurenine and 3-hydroxy anthranilic acid have potent reactive oxygen species (ROS)-scavenging anti-ferroptotic activity [11]. A recent study revealed that IDO1 improved hepatocytes ferroptosis and contributed to inflammatory hepatic damage [12]. These observations indicated a connection (that remains functionally and mechanistically controversial) between IDO1 and the execution of ferroptosis, which is a unique form of non-apoptotic cell death caused by excessive iron-dependent lipid peroxidation.

This study revealed that IDO1 expression increased GBM progression by suppressing ferroptotic cell death. IDO1 overexpression reduced the lipid peroxidation production and oxidative stress levels in GBM cells. IDO1 elevated m6A methylation, SLC7A11 mRNA stability, and AhR expression. Mechanistically, IDO1 blocked the SLC7A11-mediated ferroptosis of GBM cells through the transcriptional inhibition by which the Kyn-AhR axis regulates FTO gene expression. Our results emphasize the significance of depressing the IDO1-Kyn-AhR axis to activate ferroptosis signaling in the central nervous system as a therapeutic strategy to delay glioblastoma progression.

## Results

### IDO1 expression was upregulated in patients with GBM

**Fig. 1:**
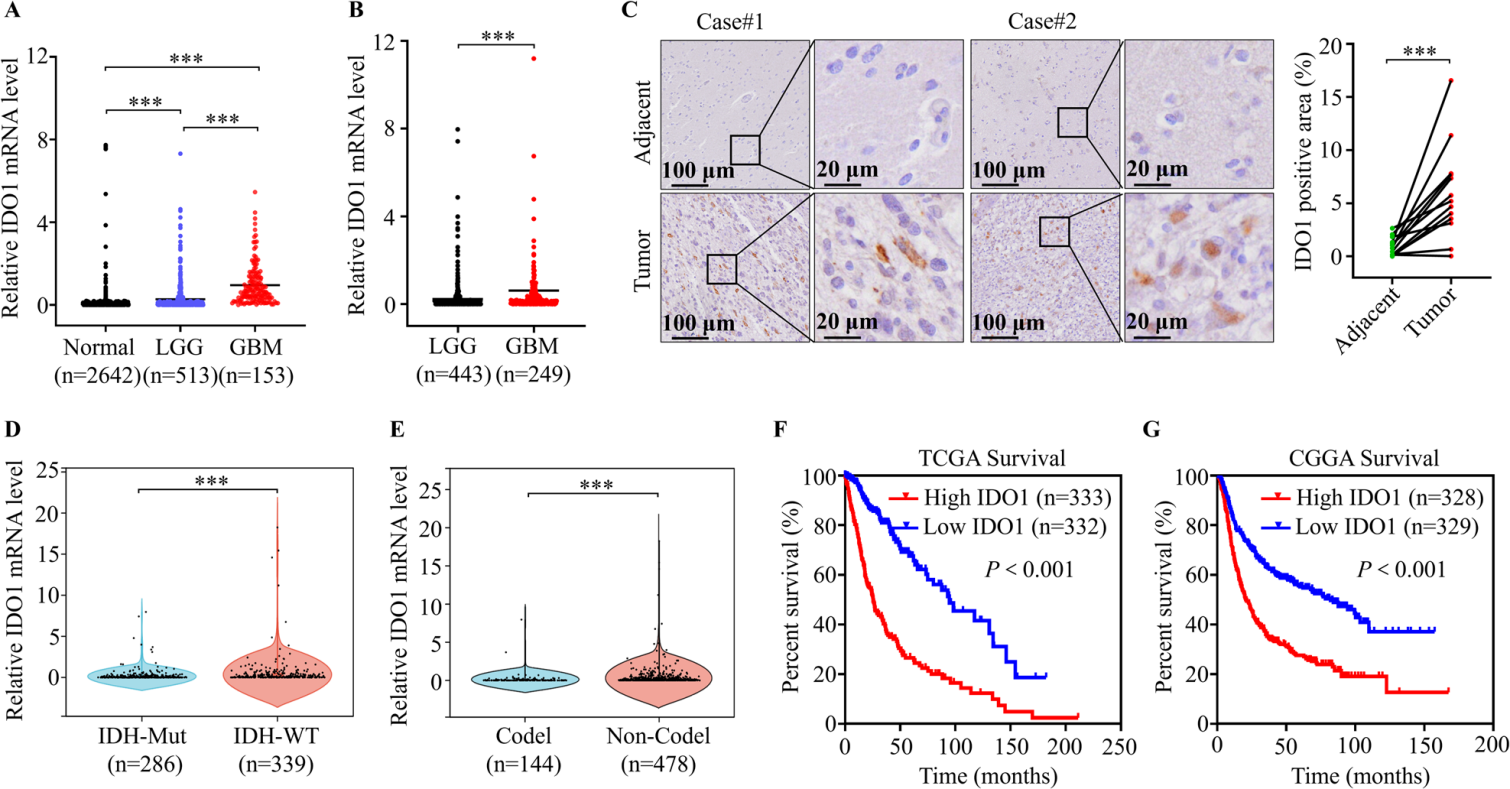
IDO1 expression was upregulated in patients with GBM. **A** The mRNA levels of IDO1 were compared between normal brain tissues (*n* = 2642), low-grade gliomas (LGG) (*n* = 513) and high-grade gliomas (GBM) (*n* = 153) tissues in the TCGA database. **B** The mRNA levels of IDO1 were compared between LGG (*n* = 443) and GBM (*n* = 249) tissues in CGGA database. **C** Immunohistochemistry (IHC) staining was used to detect the expression of IDO1 in GBM tissues and adjacent normal tissues from the clinical GBM specimens (*n* = 12), scale bar = 100 μm or 20 μm. Correlation of IDO1 mRNA levels in gliomas and isocitrate dehydrogenase (IDH) genetype (**D**) or 1p/19q codeletion status (**E**) was analyzed in CGGA database. Kaplan–Meier curves of overall survival (OS) of glioma patients from TCGA database (**F**) and CGGA database (**G**) were determined by log-rank test. The data are represented as the mean ± standard deviation (SD); *p < 0.05, **p < 0.01, ***p < 0.001, ns not significant.

We compared the mRNA levels of IDO1 among high-grade (GBM) and low-grade (LGG) glioma downloaded from The Cancer Genome Atlas (TCGA) database, and health tissues (normal) downloaded from the Genotype-Tissue Expression (GTEx) database to illustrate the biological role of IDO1 in GBM progression. We observed markedly higher IDO1 expression in patients with GBM than in those with LGG and normal tissues (Fig. 1A). Similar results were observed by analyzing IDO1 expressions between patients with GBM and LGG from the Chinese Glioma Genome Atlas (CGGA) database (Fig. 1B). We randomly collected human GBM specimens and adjacent non-tumor tissues and performed IDO1 immunohistochemistry (IHC) staining. The results revealed markedly increased IDO1 expression in GBM specimens compared to that in adjacent non-tumor tissues (Fig. 1C). Isocitrate dehydrogenase (IDH)-wildtype (WT) and 1p/19q non-codeletion represent worse prognostic hallmarks of glioma. Here, we revealed that the high IDO1 mRNA expression in glioma was positively correlated with IDH-WT and 1p/19q non-codeleted subtype, respectively (Fig. 1D, E). Moreover, Kaplan–Meier survival analysis of patients with glioma from TCGA and CGGA databases revealed that OS was significantly lower for patients with IDO1-high expression than for those with IDO1-low expression (Fig. 1F, G). These results indicate IDO1 as an important glioma progression regulator and demonstrate high IDO1 expression as a potential predictor of poor outcomes in GBM.

### IDO1 suppressed lipid peroxidation generation and ferroptosis in GBM cells

**Fig. 2:**
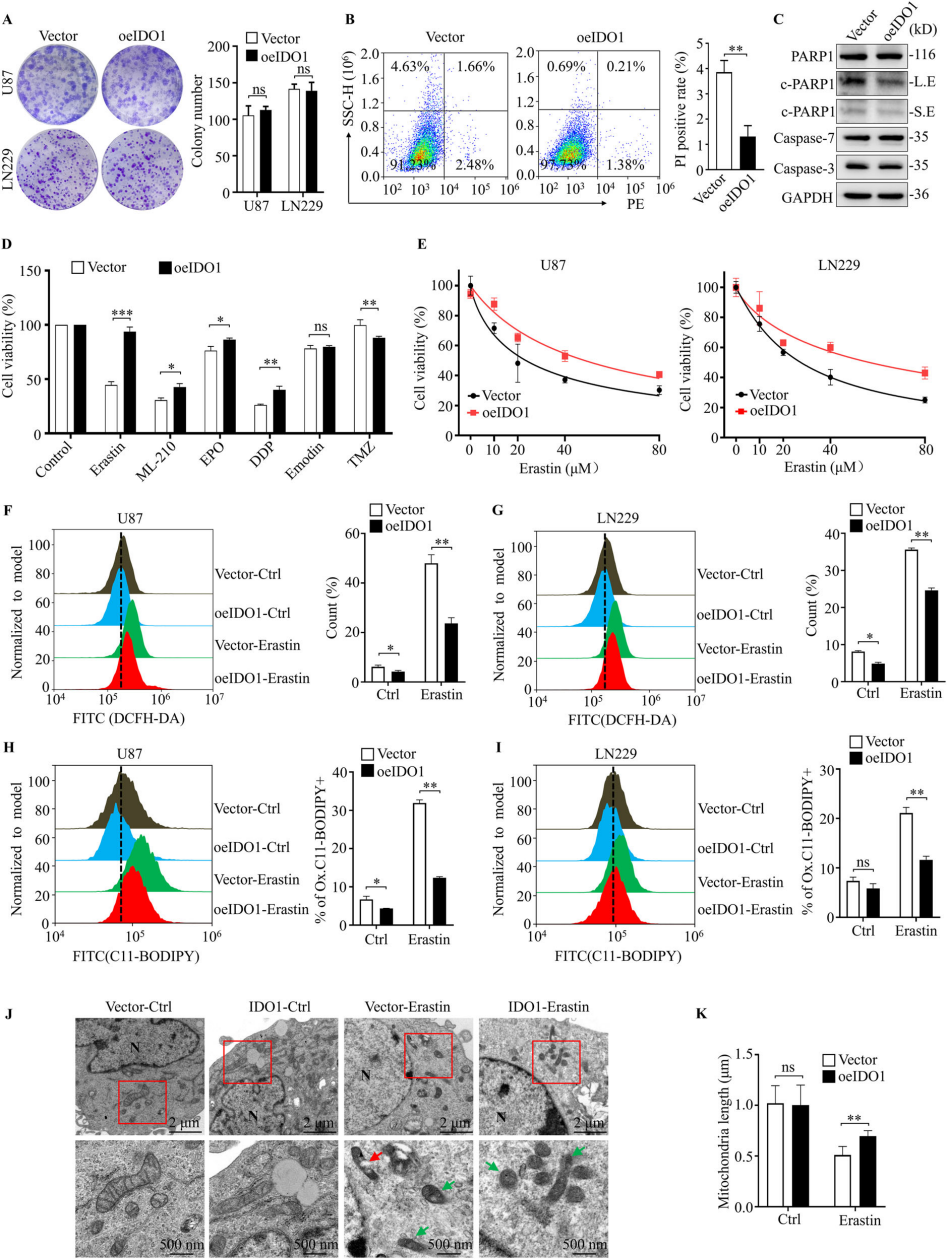
IDO1 suppressed lipid peroxidation generation and ferroptosis in GBM cells. **A** Colony formation assays were used to detect the colony seeding ability of the effects of IDO1 overexpression (oeIDO1) in U87 and LN229 cells (left), quantification of relative clones (right). **B** Cell death rate was determined by Propidium Iodide (PI) staining flow cytometry in U87 cells with IDO1 overexpression. **C** Western blotting was used to detect the expression of PARP1, cleaved-PARP1 (c-PARP1), Caspase-7, and Caspase-3 in IDO1 overexpressed U87 cells, LE: long exposure, SE: short exposure (*n* = 3 biologically replicates). **D** The cell viability of U87 cell treated with Erastin (20 μM), ML-210 (40 μM), Etoposide (10 μM), Cisplatin (20 μM), Emodin (40 μM), Temozolomide (400 μM) for 72 h. **E** The cell viability curve of U87 and LN229 cells treated with 0, 10, 20, 40, and 80 μM Erastin for 72 h. ROS was measured by flow cytometry using DCFH-DA staining in U87 (**F**) and LN229 (**G**) cells after treatment with or without Erastin. Lipid peroxidation was measured by flow cytometry using C11-BODIPY^581/591^ staining in U87 (**H**) and LN229 (**I**) cells treated with or without Erastin. **J** Representative images of typical ferroptosis mitochondrial morphology in U87 cells. Green arrows: shrunken and heavy stained mitochondria; red arrows: ruptured mitochondrial membrane; N: nucleus, scale bar = 2 μm or 500 nm. **K** The statistic results of mitochondrial length in the Vector (*n* = 6) and oeIDO1 (*n* = 6) U87 cell group. The data are represented as the mean ± standard deviation (SD); *p < 0.05, **p < 0.01, ***p < 0.001, ns, not significant.

Lentiviral-mediated IDO1 overexpression was conducted to investigate the potential oncogenic role of IDO1 in U87 and LN229 cells because GBM cells barely express IDO1 (Fig. S1A, B). Intriguingly, IDO1 overexpression significantly decreased the proliferation of both GBM cells (Fig. S1C). Further, EdU cell proliferation assay and flow cytometry revealed that IDO1 overexpression inhibited DNA synthesis and arrested the cell cycle at the S phase (Fig. S1D–I). Notably, IDO1 expression exhibited no obvious effect on the colony-seeding ability of U87 and LN229 cells (Fig. 2A). Resisting programmed cell death is one of the distinguishing hallmarks of cancers, considering that tumorigenesis is the combined effect of tumor cell proliferation and cell death. We investigated the effect of IDO1 on cell death in GBM cells and indicated that IDO1 markedly reduced the GBM cell death rate in IDO1-overexpressed U87 cells (Fig. 2B). Western blotting assay further confirmed this finding, demonstrating that DO1 overexpression decreased cleaved-PARP1 (c-PARP1, a cell death marker) expression, but not caspase-3/7, as compared with their control vector counterparts (Fig. 2C). These results indicated that IDO1 induced cell death resistance in GBM cells. Additionally, its existence reduced GBM cell proliferation, but the tumor promotion effect of IDO1 was caused by the pro-cancer ability to resist the cell death program surpassing the effect of cell proliferation inhibition.

We then investigated the effects of IDO1 on the cell death pathway sensitivity of GBM cells. We revealed the markedly decreased cell death of IDO1 overexpression on GBM cells by the ferroptosis inducers (Erastin, ML-210), moderately by the apoptosis inducers (EPO and DDP), and hardly by the necrosis inducers (Emodin and TMZ). Further, the resistance of erastin, among these cell death inducers, was the most significant in IDO1 overexpressed cells (Fig. 2D). The half maximal inhibitory concentration (IC_50_) of erastin was increased from 24.0 μM to 48.6 μM in IDO1 overexpressed U87 cells. A similar result was obtained in LN229 cells that the IC_50_ of erastin was increased from 27.4 μM to 56.5 μM during IDO1 overexpression (Fig. 2E). Notably, IDO1 overexpression in U87 and LN229 cells significantly blocked erastin-induced cytosolic ROS production, as detected by flow cytometry using fluorescent probe DCFH-DA (Fig. 2F, G). Lipid peroxidation levels were further compared using lipid peroxide sensor C11-BODIPY^581/591^ and IDO1 overexpression decreased the lipid peroxide generation in GBM cells treated with or without erastin (Fig. 2H, I). In addition, transmission electron microscopy showed typical ferroptosis mitochondrial morphology, including shrunken and darker stained mitochondria (green arrow), ruptured mitochondrial membrane (red arrow) and normal nucleus without chromosome condensation (N) in the erastin-treated U87 cells. However, IDO1 overexpression decreased mitochondrial damage, appeared as less stained mitochondria and few ruptured mitochondria (Fig. 2J). Consistently, quantitative analysis of mitochondrial length showed that, compared to IDO1-overexpressed cells, the mitochondria was significantly smaller in Vector cells with erastin treatment (Fig. 2K). Moreover, enzyme-linked immunosorbent assay (ELISA) revealed that IDO1 overexpression significantly diminished the abundance of malondialdehyde (MDA) and intracellular Fe^2+^ compared to control cells, although the Fe^2+^ levels failed to show statistical difference (Fig. S1J). These results indicate the important role played by IDO1 in executing ferroptosis in GBM cells.

### IDO1 regulation of ferroptosis in GBM cells depends on SLC7A11 expression

**Fig. 3:**
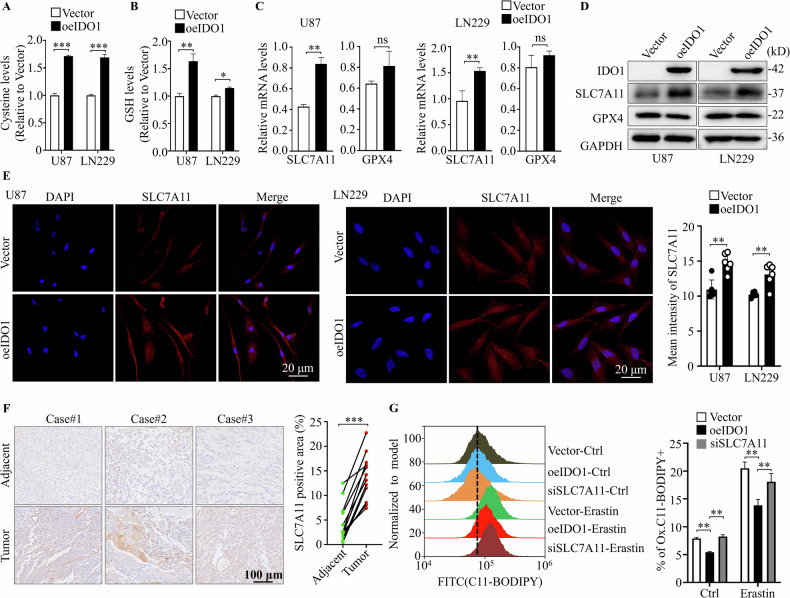
IDO1 regulation of ferroptosis in GBM cells depends on SLC7A11 expression. Cystine levels (**A**) and glutathione (GSH) levels (**B**) were detected in U87 and LN229 cells with IDO1 overexpression. **C** RT-qPCR was used to detect the mRNA levels of IDO1, SLC7A11 and GPX4 in U87 and LN229 cells with IDO1 overexpression (*n* = 3 biologically replicates). **D** Western blotting was used to detect the protein expression of IDO1, SLC7A11, and GPX4 in U87 and LN229 cells with IDO1 overexpression (*n* = 3 biologically replicates). **E** Immunofluorescence (IF) staining showed the protein expression and subcellular distribution of SLC7A11 in U87 and LN229 cells with IDO1 overexpression, scale bar = 20 μm. **F** IHC staining was used to detect the expression of SLC7A11 in GBM tissues and adjacent normal tissues from the clinical GBM specimens (*n* = 12), scale bar = 100 μm. **G** Lipid peroxidation levels were measured by C11-BODIPY^581/591^ staining flow cytometry after SLC7A11 knockdown in U87 cells. The data are represented as the mean ± standard deviation (SD); *p < 0.05, **p < 0.01, ***p < 0.001, ns not significant.

Cellular cystine and subsequent glutathione (GSH) levels modulate the cell sensitivity to ferroptosis induced by lipid peroxidation. ELISA assays revealed significantly increased cystine and GSH levels when IDO1-overexpressed in GBM cells (Fig. 3A, B). SLC7A11 functions as an antiporter for cystine/glutamate to facilitate the synthesis of GSH, and glutathione peroxidase 4 (GPX4) acts as an enzyme that catalyzes lipid hydroperoxide reduction so that protects cells against ferroptosis. Quantitative reverse transcription polymerase chain reaction (RT-qPCR) and Western blotting analysis revealed that IDO1 overexpression increased the mRNA and protein expression levels of SLC7A11, but not GPX4, in GBM cells (Fig. 3C, D). Immunofluorescence (IF) staining was conducted to further visualize the changes of expression and the subcellular distribution of SLC7A11 accompanying IDO1 overexpression in U87 and LN229 cells (Fig. 3E). Similarly, IHC results revealed that SLC7A11 expression in GBM tumors was significantly higher compared to that in adjacent non-tumor tissues (Fig. 3F). Importantly, siRNA-mediated SLC7A11 knockdown (siSLC7A11) effectively rescued the reduction of lipid peroxidation generation caused by IDO1 overexpression in U87 cells (Fig. 3G). Our data indicate that suppressed ferroptosis of IDO1-proficient GBM cells is caused at least partly by upregulating SLC7A11 expression.

### IDO1 increased the SLC7A11 mRNA stability by increasing m6A deposition

**Fig. 4:**
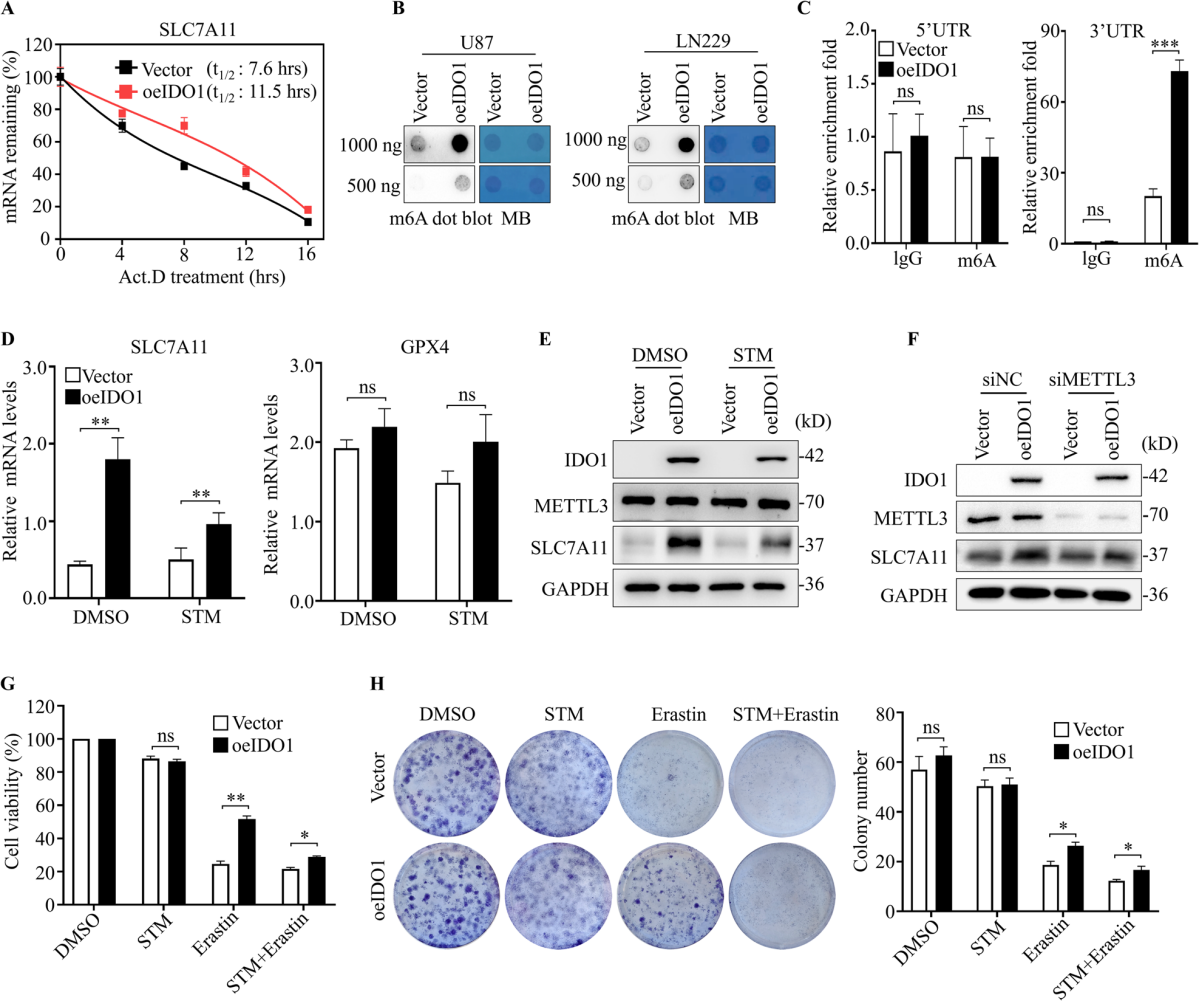
IDO1 increased the SLC7A11 mRNA stability by increasing m6A deposition. **A** SLC7A11 mRNA half-lives (t_1/2_) were determined by RNA decay rates detected by RT-qPCR in U87 cells. Data were collected at indicated timepoints with Actinomycin D (Act. D) treatment (*n* = 3 biologically replicates). **B** The m6A levels of total RNA in U87 and LN229 cells were determined by m6A dot blot. **C** MeRIP-qPCR analysis was used to determine m6A levels at indicated sites within SLC7A11 mRNA sequence in U87 cells with IDO1 overexpression (*n* = 3 biologically replicates). **D** RT-qPCR was used to detect the transcriptional levels of SLC7A11 and GPX4 in IDO1 overexpressed U87 cells with or without STM2457 (STM) treatment for 72 h (*n* = 3 biologically replicates). Western blotting was used to detect the expression of IDO1, SLC7A11 and METTL3 in U87 cells with IDO1 overexpression with STM2457 treatment for 72 h **E** or METTL3 knockdown **F** (*n* = 3 biologically replicates). **G** CCK-8 assay was used to detect the cell viability treated with Erastin (20 μM) combined with or without STM2457 (5 μM) for 72 h in U87 cells with IDO1 overexpression. **H** Colony formation assay was used to detect the colony seeding ability of cells treated with Erastin (5 μM) combined with or without STM2457 (5 μM) for 12 days in U87 cells with IDO1 overexpression. The data are represented as the mean ± standard deviation (SD); *p < 0.05, **p < 0.01, ***p < 0.001, ns not significant.

We then investigated the molecular mechanism by which IDO1 upregulates SLC7A11 expression. A luciferase reporter assay revealed that IDO1 expression did not affect the promoter activity of the SLCA711 gene (Fig. S2A), indicating that IDO1 regulates SLCA711 expression through a post-transcriptional mechanism. We conducted an mRNA stability assay in vector and IDO1-overexpressed U87 cells treated with 5 μg/ml of Act.D, a transcriptional inhibitor, to substantiate this hypothesis. As expected, the half-life of SLC7A11 mRNA was prolonged from 7.6 h to 11.5 h in IDO1 overexpressed U87 cells (Fig. 4A). These results indicate that IDO1 strongly upregulates SLC7A11 expression by improving SLC7A11 mRNA stability.

Moreover, m6A methylation is the best-characterized RNA modification and is involved in almost all mRNA life cycle stages, including splicing, export, and stability. Emerging evidence indicates the involvement of m6A modification in regulating proliferation, invasion, stemness maintenance, and radiation resistance of GBM cells. Dot blot assays revealed significantly increased global m6A methylation levels when IDO1 was overexpressed in U87 and LN229 cells (Fig. 4B). These observations helped us to speculate the effects of IDO1 on the mRNA stability of SLC7A11 by improving m6A deposition. We predicted the m6A modification sites of the SLC7A11 mRNA via SRAMP (http://www.cuilab.cn/sramp/), which is a sequence-based m6A modification site predictor. The analysis revealed that numerous positions in the mRNA sequences of SLC7A11 may be m6A modification sites, and the prediction scores for >2 of the positions were distributed in the high- or very high-confidence regions (Fig. S2B). We designed PCR primers to amplify an approximately 100-bp sequence that involved the very high-confidence site (around site 1795 bp) in the 3’UTR region, and one in the 5’UTR region as the negative control. MeRIP-qPCR assay revealed that the m6A methylation on the 3’UTR region of SLC7A11 mRNA, but not on the 5’UTR region, was significantly increased in vector U87 cells. Importantly, IDO1 overexpression further improved the m6A modification of the 3’UTR region (Fig. 4C). Consistently, m6A inhibitor (STM2457) treatment reversed SLC7A11 mRNA upregulation and protein expression in IDO1-overexpressed cells but did not affect GPX4 mRNA level (Fig. 4D, E). Similarly, METTL3 knockdown markedly decreased SLC7A11 protein expression in IDO1-overexpressed U87 cells (Fig. 4F). Further cell functional experiment analysis revealed that STM2457 treatment observably reversed the resistance of erastin-induced cell death in IDO1-overexpressed cells (Fig. 4G, H). Altogether, we first reveal that IDO1 controls the ferroptosis and mRNA stability of the SLCA711 gene through an m6A-related regulatory mechanism in GBM cells.

### IDO1 promoted m6A methylation via AhR-mediated transcriptional inhibition of the FTO gene

**Fig. 5:**
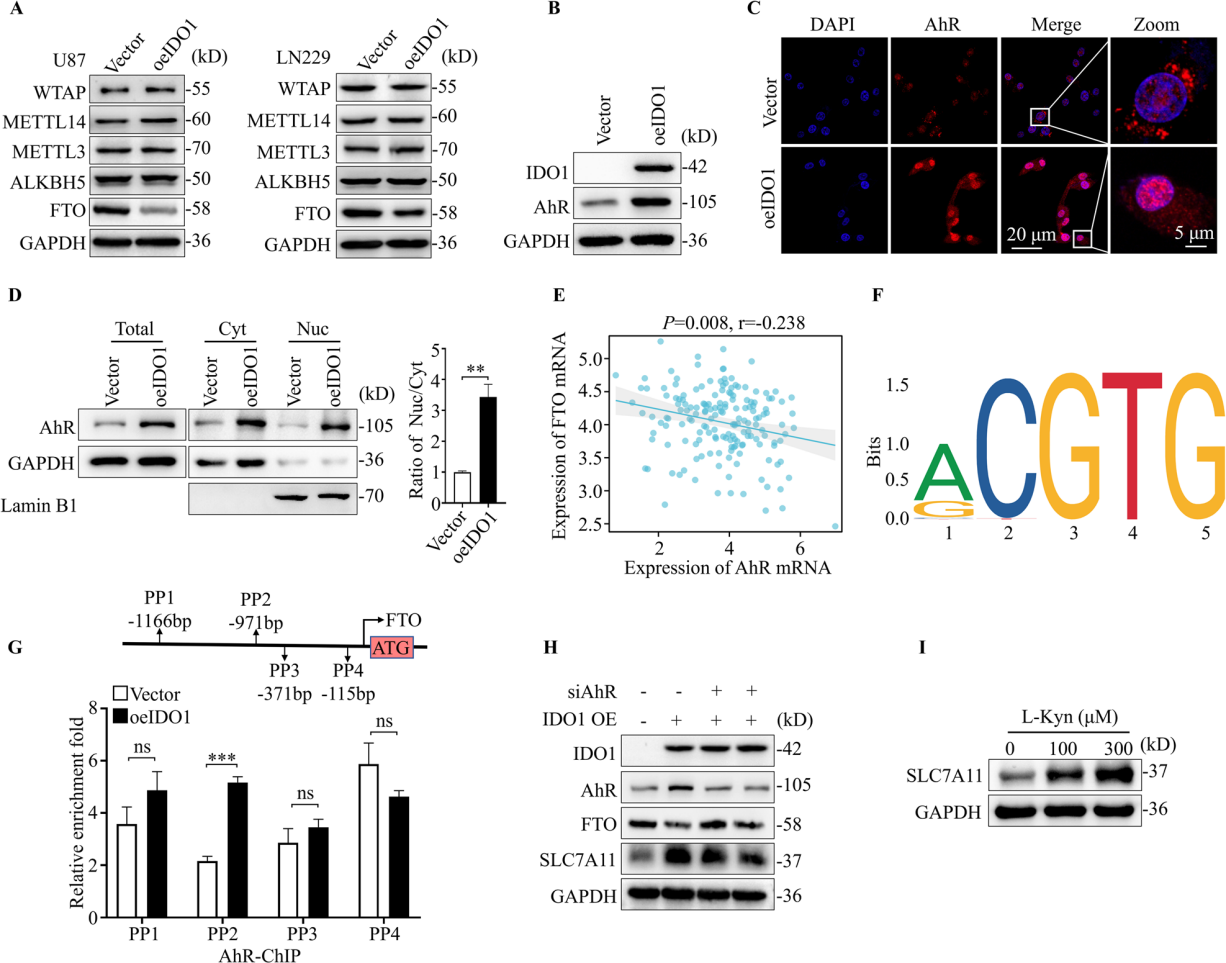
IDO1 promoted m6A methylation via AhR-mediated transcriptional inhibition of the FTO gene. **A** Western blotting was used to detect the expression of m6A regulators in U87 and LN229 cells with IDO1 overexpression (*n* = 3 biologically replicates). **B** Western blotting was used to detect the expression of IDO1 and AhR in U87 cells with IDO1 overexpression (*n* = 3 biologically replicates). **C** IF was used to visualized subcellular distribution of AhR in U87 cells with IDO1 overexpression. scale bar = 20 μm or 5 μm. **D** Western blotting analysis of the expression of AhR in total, cytoplasmic (Cyt), and nuclear (Nuc) protein samples of U87 cells with IDO1 overexpression. Cytoplasmic GAPDH and nuclear Lamin B1 were used as controls for normalization (*n* = 3 biologically replicates). **E** Correlation between the expression of FTO and AhR in clinical GBM samples was analyzed by transcriptome profiling in the TCGA glioblastoma database. **F** Predicted FTO binding motifs (CGTG) were obtained using the JASPAR public database. **G** Schematic diagram of potential AhR binding sites in the promoter region of FTO, and ChIP-qPCR analysis was used to detect the enrichment of AhR on FTO promoter (*n* = 3 biologically replicates). **H** Western blotting was used to evaluate the protein levels of IDO1, AhR, FTO, SLC7A11 after AhR knockdown in IDO1 overexpressed U87 cells (*n* = 3 biologically replicates). **I** Western blotting analysis of the expression of SLC7A11 in U87-WT cells treated with different concentrations of Kyn for 48 h (*n* = 3 biologically replicates). The data are represented as the mean ± standard deviation (SD); *p < 0.05, **p < 0.01, ***p < 0.001, ns, not significant.

Cellular RNA m6A methylation is identified by the dynamic regulation of m6A regulators, including methyltransferase complex (METTL3/METTL14/WTAP) and demethylase (FTO and ALKBH5). Here, we revealed that IDO1 overexpression decreased FTO protein expression but did not affect other m6A regulator expression (Fig. 5A), indicating that m6A accumulation caused by IDO1 overexpression was attributed to demethylase expression downregulations, such as FTO rather than methyltransferases in GBM cells.

We further investigated the molecular mechanism by which IDO1 downregulates FTO expression. Reportedly, AhR, a considerable downstream ligand-dependent transcription factor of IDO1, positively regulated FTO expression in Leydig cells. We revealed that AhR protein expression was markedly increased in IDO1-overexpressed cells compared to that in vector U87 cells (Fig. 5B). Accordingly, IF staining and subcellular component separation assays revealed that IDO1 overexpression increased the AhR protein expression and nuclear distribution (Fig. 5C, D). Further, the correlation analysis of AhR and FTO expression in 169 glioblastoma samples revealed that AhR expression was negatively correlated with FTO expression (R = −0.238, *p* = 0.008) (Fig. 5E), indicating that IDO1 inhibits FTO activity by promoting AhR expression and nuclear translocation. Previous studies have demonstrated that AhR regulates gene expression by directly binding to the promoter regions of its target genes. We suspect that IDO1 suppresses FTO transcriptional activation by affecting AhR recruitment in the promoter regions. To confirm, we obtained the AhR binding motif (CGTG) from the JASPAR database (Fig. 5F). Chromatin immunoprecipitation (ChIP)-qPCR result indicated that AhR specifically binds to the four predicted promoter regions of the FTO gene, and overexpression of IDO1 significantly improved AhR binding levels (around site -971bp) in U87 cells (Fig. 5G). Knockdown by siRNA, directed against AhR, increased FTO protein expression while decreasing the SLC7A11 expression in IDO1-overexpressed U87 cells (Fig. 5H). Finally, we revealed that IDO1 downstream metabolite Kyn (an active AhR ligand) improved the SLC7A11 expression in U87 cells, which was consistent with the effect of IDO1 overexpression (Fig. 5I). These results indicate that IDO1 downregulates the gene expression of FTO through an AhR-mediated transcriptional inhibition mechanism, thereby preventing m6A accumulation.

## Discussion

Ferroptosis is a novel form of iron-dependent regulated cell death distinct from apoptosis, necrosis, autophagy, and other cell death mechanisms. This unique cell death type is caused by culminating overwhelming oxidative stress and lipid peroxidation downstream of metabolic dysfunctions [13, 14]. The solute carrier family 7 member 11 (SLC7A11, also known as xCT) is the central antiporter involved in the ferroptosis suppression necessary for cystine import and subsequent antioxidant GSH biosynthesis [15, 16]. Our study revealed that IDO1 overexpression decreased cell death and increased mRNA and protein SLC7A11 levels in GBM cells. We determined significantly higher SLC7A11 expression in GBM tissues than in normal tissues. Moreover, SLC7A11 knockdown effectively rescued lipid peroxidation level reduction in IDO1 overexpressed GBM cells. These results revealed the regulatory mechanism of developing an anti-ferroptosis defense by upregulating SLC7A11 expression as a key consequence of IDO1 activity in GBM cells, thereby reducing cell death and promoting tumor progression.

SLC7A11 is recognized as a substantial factor of a ferroptosis suppressor, and transcription factors and epigenetic mechanisms regulate its expression [17]. Our results indicated that IDO1 regulated SLC7A11 expression via epigenetic modifications in GBM cells. The m6A modification is widespread throughout the transcriptome and critical in epigenetic regulation [18]. Accumulating evidence indicates that the m6A modification participates in GBM carcinogenesis. In particular, it regulates GBM cell proliferation, invasion, angiogenesis, stemness maintenance, chemotherapy sensitivity, and radiation resistance, representing a promising target for GBM treatment [19, 20]. The present results indicated that IDO1 regulates SLC7A11 expression via a m6A modification mechanism by affecting SLC7A11 mRNA stability. We confirmed that IDO1 increases m6A modification level, and SLC7A11 mRNA undergoes aberrant m6A modification in the 3’UTR region, which improves SLC7A11 mRNA stability. Thus far, the m6A reader proteins, including IGF2BP1-3, YTHDF2, YTHDF3, and YTHDC2, have bound to the m6A modification of mRNA and regulate its stability [21, 22]. Future studies are warranted to further identify the reader protein, which recognizes and binds to the m6A modification of SLC7A11 mRNA to upregulate SLC7A11 expression in GBM cells.

**Fig. 6:**
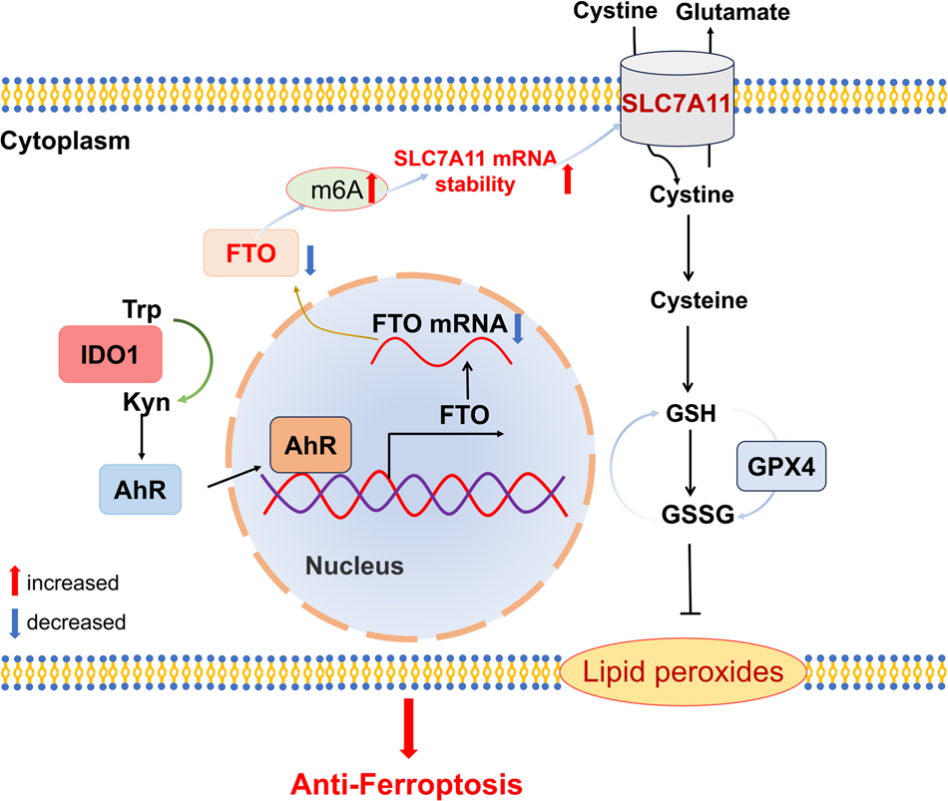
Schematic diagram of IDO1-AhR-FTO axis inhibits GBM ferroptosis in an m6A-SLC7A11-mediated manner. IDO1 promoted the AhR expression and nuclear translocation, thus facilitating AhR recruitment at the promoter regions of FTO gene and negatively regulating its transcription. FTO downregulation leads to accumulation of the m6A deposition to improve SLC7A11 mRNA stability and expression, thereby inhibiting ferroptosis in GBM cells.

The m6A modification levels are identified by the interplay between methyltransferases and demethylases. Our work confirmed that IDO1 overexpression decreased FTO expression but did not affect other m6A regulators’ expression, indicating that IDO1-induced m6A accumulation was related to demethylase downregulations, rather than methyltransferases in GBM cells. FTO has been involved in various biological processes, including cell proliferation, migration, and apoptosis [23]. A previous study in reproductive cells indicated that Bisphenol F (BPF) positively regulated FTO through AhR activation [24]. AhR is a ligand-dependent basic helix-loop-helix transcription factor that is activated by various ligands, including environmental contaminants (such as pollutant BPF and dioxin) and endogenous ligands (such as TRP metabolites, dietary components, and indigoids) [25]. AhR undergoes conformational changes and translocates into the nucleus after binding with its specific ligands, then binds to the xenobiotic response element in the promoter region of target genes, thereby exerting various regulatory effects, including gene transcription activation, as well as gene transcription inhibition [24, 26]. We propose a model based on the experimental data we obtained above to demonstrate how IDO1-Kyn-AhR axis negatively regulates the FTO expression and subsequently increases the m6A deposition to improve SLC7A11 mRNA stability and expression, thereby inhibiting ferroptosis in GBM cells (Fig. 6).

The present study indicates that IDO1 acts as an oncogene by suppressing the ferroptotic cell death pathway and promoting tumor progression in patients with GBM. This pro-cancer effect may predominantly exist and widely contribute to the poor tumor prognosis, considering the high T-cell infiltration and IDO1 expression levels in GBM cells. Our results indicate that m6A inhibition decreases the ferroptosis suppressor SLC7A11 expression and reverses ferroptotic cell death resistance in GBM cells. This research provides a strategy to downregulate anti-ferroptosis pathway activity and indicates that m6A may serve as a potential target for improving anti-GBM therapy.

## Materials and methods

### Cell culture, transfection, and IDO1 overexpression cell line establishment

Glioblastoma (GBM) U87 and LN229 cell lines were obtained from the Cell Bank/Stem Cell Bank of the Chinese Academy of Sciences (Shanghai, China). Cells were maintained in 4.5 g/L high-glucose DMEM medium (Gibco, USA) supplemented with 10% fetal bovine serum (FBS, China), 1% penicillin and 1% streptomycin, and cultured in a constant temperature incubator at 37 °C and 5% CO_2_. Cell line authenticity was confirmed through STR profiling, and tested for mycoplasma contamination regularly. Transfection was performed with Lipofectamine 3000 transfection reagent (L3000015, Invitrogen, USA) according to the manufacturer’s instructions. The interfering RNAs (siRNAs) targeting SLC7A11, AhR and METTLE3 were obtained from Sangon Biotech (Shanghai, China), and siRNA sequences were showed in Supplementary Table 1. The IDO1 overexpression lentivirus and the corresponding control lentivirus were obtained from Hanbio (Shanghai, China). Lentiviruses were mixed with serum-free medium using 4 μg/ml polybrene (HB-PB-500, Hanbio, China) and added to the GBM cells, then replaced with complete medium 6–8 h later. After 48 h of cell culture, 1 μg/ml of puromycin (P8230, Solarbio, China) was added to the medium to screen the cells for 7 days to establish IDO1 overexpression cell lines.

### Human ethics and organization collection

Informed consent was obtained from each patient prior to the acquisition and using the clinical GBM specimens through a written agreement, which was approved by the Ethics Committee of Guizhou Medical University. A total of 12 pairs of GBM tissue samples and there corresponding normal brain tissues were obtained from patients who underwent surgical resection therapy.

### Cell proliferation detection

Cell proliferation was determined using a Cell Counting Kit-8 (CCK-8) kit (K1018, APExBIO, USA). GBM cells were seeded in 96-well plates at a density of 5 × 10^3^ cells/well and cultured for 1, 2, 3 days. Then, 10 μl CCK8 reagent was added to each well and incubated at 37 °C for 2 h. The absorbance (OD value) at 450 nm was measured and relative cell proliferation was calculated according to the OD values. For 5-ethynyl-2’-deoxyuridine (EdU) cell proliferation assay, cells were inoculated in a 6-well plate at a density of 1 × 10^5^ cells/well, then performed the experiment at the following day using the BeyoClick™ EDU-555 cell proliferation kit (C0075S, Beyotime, China) according to the manufacturer’s instructions. Images were captured using a confocal microscope (Olympus SpinSR 10).

### Cell viability

U87-Vector (control group) and U87-oeIDO1 (IDO1 overexpression group) cells were seeded in triplicate on 96-well plates and treated with different cell death inducing compounds the next day, including 20 μM Erastin, 400 μM Temozolomide (TMZ), 10 μM Etoposide (EPO), 20 μM Cisplatin (DDP), 40 μM ML-210 and 40 μM Emodin for 72 h. Cell viability was assessed using CCK-8 method and results were normalized by the control group (DMSO treatment group). The half maximal inhibitory concentration (IC_50_) was calculated by the cell viability treated with a series of concentrations of Erastin for 72 h and cell proliferation curve was plotted using GraphPad Prism 8.0 software.

### Western blotting

Cell proteins were extracted with RIPA lysis buffer (P0013B, Beyotime, China), 1 mM phenyl methyl sulfonyl fluoride (PMSF, P0100, China), and 1× phosphatase inhibitor (Roche, Basel, Switzerland). The protein concentration from each group was determined with BCA kit (PC0020, Solarbio, China). A total of 20 μg protein was loaded on a sodium dodecyl sulfate polyacrylamide gel electrophoresis (SDS-PAGE), transferred to a polyvinylidene difluoride (PVDF) membrane and blocked with 5% non-fat milk for 1 h. The membrane was incubated with the primary antibodies listed in Supplementary Table 2. The imprinted images were captured by Tanon Imaging System (TANON-5200, China), and density was determined by ImageJ software. All experiments were performed for at least three biological replicates and representative images were displayed in the corresponding Figures.

### Immunohistochemistry (IHC) staining

The harvested tumor samples were encased in paraffin and cut into 5 μm thick tissue slices following a routine procedure. After fully dewaxed and rehydrated, the tissue sections were recalledin 3% hydrogen peroxide to block endogenous peroxidase activity. The tissue sections were blocked with 5% fetal bovine serum albumin (BSA) at room temperature for 1 h, and placed flat in a wet box and incubated with primary antibodies listed in Supplementary Table 2 at 4 °C overnight. Subsequently, the sections were washed three times with PBS and covered with immunohistochemical secondary antibody at 37 °C for 20 min, followed by DAB staining, hematoxylin staining and then imaged under a microscope. The results were evaluated and analyzed in a blinded method by two independent pathologists.

### Immunofluorescence (IF) assay

Appropriate amount of cells (2 × 10^5^) were seeded in 6-well plates, fixed with 4% paraformaldehyde (PFA) for 15 min on the second day, treated with 0.3% TritonX-100 for 10 min, and then blocked using 3% BSA solution. Primary antibodies against AhR (28727-1-AP, proteintech, China), SLC7A11 (12691, Cell Signaling Technology, USA) were incubated overnight at 4 °C. This procedure was followed by incubation with a secondary immunofluorescence antibody (33112ES60, Yeasen, China) for 1 h after washing three times with PBS. Images were captured using a confocal microscope (Olympus SpinSR 10).

### Reverse transcription-quantitative polymerase chain reaction (RT-qPCR)

Total RNA from GBM cells was extracted using TRIzol reagent and reverse-transcribed to cDNA using an mRNA reverse transcription Kit (RR036A, Takara, Japan) according to the manufacturer’s protocol. Subsequently, qPCR was performed with TB Green® Premix (RR820A, Takara, Japan) on a BIO-RAD CFX96TM Real-Time system with the specific primers listed in Supplementary Table 1. The experiment was repeated at least three times, and the relative mRNA expression was determined using the 2^-∆∆Ct^ method.

### Cell cycle assay and PI cell death staining

The cell cycle assay was performed according to the kit instructions (KGA 512, Keygen, China). In briefly, cells were washed with pre-cooled PBS, centrifuged at 800 rpm for 5 min, and fixed with pre-cooled 70% ethanol overnight at 4 °C. Cells were washed with PBS and treated with 20 mg/ml RNase A for 30 min, then stained with propyl iodide (PI) solution in the dark at room temperature for 15 min. Cell cycle analysis was performed by flow cytometry (ACEA NovoCyte Fluidics Station, BD Biosciences, USA). For PI cell death staining assay, cells were carefully harvested by trypsin digestion, washed with PBS, stained with 20 μg/mg PI (C0080, Solarbio, China) and immediately determined by flow cytometry method. All data were analyzed using FlowJo 7.4.1 software.

### Lipid peroxidation and ROS level detection

Cells were digested, centrifuged at 800 rpm for 5 min and washed twice with PBS before detection. For lipid peroxidation assay, cells were stained with 5 μM of C11-BODIPY^581/591^ (D3861, Invitrogen, USA) at 37 °C for 30 min, and detected by flow cytometry method (excitation: 488 nm, emission: 510 nm). For ROS assay, cells were stained with 10 μM of 2′,7′-dichlorodihydrofluorescein diacetate (DCFH-DA, S0033S, Beyotime, China) and assessed by flow cytometry (excitation: 488 nm, emission: 510 nm). Data were analyzed by flow cytometer software (ACEA NovoCyte Fluidics Station, BD Biosciences, USA).

### Ferroptosis detection

GBM cells were digested by trypsin, collected by centrifugation and washed twice with PBS before detection. Glutathione (GSH) Assay kit (A006-2-1, Nanjing Jiancheng, China) was employed according to the manufacturer’s instructions to assess the GSH levels. Cysteine (Cys) assay kit (JN24232, JINING Industrial, China) was used to measure intracellular Cys levels in accordance with the manufacturer’s protocols. The malondialdehyde (MDA) levels were detected using the Lipid Peroxidation (MDA) Assay Kit (BC0025; Solarbio, China), while the Fe^2+^ concentrations were assessed with an Iron Assay Kit (E-BC-K881-M, Elabscience, China) in U87 and LN229 cells.

### Transmission electron microscopy

Cell precipitates were collected after centrifugation. The sample was prefixed with 2.5% glutaraldehyde, refixed with 1% osmium tetroxide, and precipitated in the fixative. Next, cells were dehydrated through an acetone series. The further dehydrating agent and Epon-812 embedding agent were infiltrated according to the proportions of 3:1, 1:1 and 1:3, respectively. Then, after embedding with Epon-812 pure embedding agent, the 60–90 nm ultra-thin slice was made by ultra-thin microtome and retrieved into copper net. Then Dyed with uranium acetate for 10–15 min, and stained with lead citrate for 1–2 min. Transmission electron microscope (JEM-1400FLASH, Nippon Electronics Corporation, Japan) was used to acquire images of copper mesh. The length of mitochondria was measured by ImageJ software.

### m6A dot blot assays

Total RNA was denatured at 55 °C for 5 min and placed on ice for 3 min. The denatured RNA was then transferred onto a properly sized nylon membrane (YA1760, Solarbio, China) by adding the samples to a BioDot apparatus (BJKP-KP-31B, China). After ultraviolet cross-linking for 30 min, the membranes were stained with 1% methylene blue staining buffer (G1303, Solarbio, China) for 3 min to guarantee consistency across samples. After three times wash with distilled water, the membranes were blocked with 5% non-fat milk for 1 h and incubated with m6A antibody (202003, Synaptic Systems, USA) overnight at 4 °C. Next, the membranes were incubated with secondary HRP-conjugated anti-rabbit antibody (SA00001-2, proteintech, China) for 1 h. Images were obtained using the Tanon Imaging System.

### m6A immunoprecipitation-qPCR (MeRIP-qPCR)

Total RNA was isolated from cells using TRIzol reagent. 60 μg RNA were incubated with m6A antibody (1:50, 202003, Synaptic Systems, USA) overnight on a rotator in 500 μl RIP lysis buffer (7008S, Cell Signaling Technology) at 4 °C containing 2 μl RNase inhibitor (R8061, Solarbio, China). Next, the RNA-antibody complex sample was fully mixed with 30 μl of protein G magnetic beads (9006S, Cell Signaling Technology, USA) and incubated for 2 h at 4 °C. The beads were washed three times with immunoprecipitation buffer on a magnetic rack, and the RNA was eluted two times using eluent solution. The m6A-modified RNA was purified with TRIzol reagent from the magnetic beads, followed by RT-qPCR to detect the gene expression with the primers listed in Supplementary Table 1 and the experiment was repeated at least three times. IgG served as a negative control.

### Chromatin immunoprecipitation-qPCR (ChIP-qPCR) assays

ChIP analysis was conducted with AhR (1:100, 28727-1-AP, proteintech, China) antibody following the protocol of the Simple ChIP Kit (9003S, Cell Signaling Technology, USA). Briefly, U87-Vector and U87-oeIDO1 cells (1 × 10^7^) were collected, crosslinked with 1% formaldehyde solution for 10 min and neutralized with glycine for 5 min. After incubating in buffer A for 10 min, nuclei in the cell mass was collected by centrifugation. After digestion with buffer B containing micrococcus nuclease (25 units), the cell chromatin DNA was clipped by pulsed ultrasound and harvested by centrifugation at 12,000 rpm for 10 min. Then the same amount of chromatin DNA was incubated with the primary antibody against AhR and IgG at 4°C overnight, respectively. Antibody pull-down DNA was purified by spin columns and quantitated by qPCR using the BIO-RAD CFX96TM Real-Time system with the primers listed in Supplementary Table 1. The experiment was repeated at least three times and gene expression was calculated by 2^−∆∆Ct^ method.

### RNA stability assays

U87-Vector and U87-oeIDO1 cells were treated with 5 μg/ml Actinomyctin D (Act.D) (GC16866, GIpBio, China) for 0, 4, 8, 12, 16 (h). Then, Total RNA was then extracted using TRIzol reagents and quantified using BIORAD CFX 96TM real-time fluorescent qPCR with the primers listed in Supplementary Table 1. The percentage of remaining mRNA was calculated by the amount of RNA at a specified time point relative to the RNA level at 0 h. The experiment was repeated at least three times and gene expression was calculated by the 2^-∆∆Ct^ method.

### Dual-luciferase reporter assays

The SLC7A11 promoter region sequences were inserted into the pGL4.20 luciferase reporter vector (E675A, Promega, China) and then transfected into U87-Vector and U87-oeIDO1 cells using Lipofectamine 3000 transfection reagent (L3000015, Invitrogen, USA) following the manufacturer’s protocol. Normalization and evaluation of transfection efficiency were carried out with a vector containing the pRL Renilla luciferase reporter. After 48 h of transfection, the cells were harvested and lysed with lysis buffer. The Dual-Glo Luciferase Assay System (E2920, Promega, China) was used to measure reporter activity. The firefly luciferase activity was normalized to that of Renilla for each sample well. Assays were performed in triplicate and the independent experiment was repeated at least three times.

### Prediction of m6A modification sites and AhR binding sites

SLC7A11 mRNA sequence and FTO promoter sequences were downloaded from NCBI database, SRAMP (http://www.cuilab.cn/sramp/) was used to predict the SLC7A11 mRNA m6A modification sites. And the FTO promoter sequence and binding sites by AhR were obtained through the transcription factor database (JASPAP^2024^).

### Statistical analysis

GraphPad Prism 8.0 software was used to determine statistically significant differences. No statistical methods were used to predetermine sample sizes. All experiments were carried out using at least three biological replicates. All examinations were performed on discrete samples and the results of all key experiments were reliably reproduced. Quantitative data are presented as the mean ± SD, as denoted in the figure legends. Statistical comparisons between two groups were carried out by Student’s t-test. One-way ANOVA was used for multiple-group comparisons. Survival curves were generated using the Kaplan–Meier method and compared using the log-rank test. The indicated *p* values (*p < 0.05) were considered statistically significant. Statistical details of each experiment were described in the corresponding figure legends.

## Supplementary information

Supplementary Information-Original Data

Supplementary Information-primers and antibody

## Data Availability

The datasets generated and/or analyzed during the current study are available from the corresponding author (BMJ) on reasonable request.
