# Supplementary figures and images for: IDO1 inhibits ferroptosis by regulating FTO-mediated m6A methylation and SLC7A11 mRNA stability during glioblastoma progression
Source: Cell Death Discov. 2025 Jan 25;11:22. doi: 10.1038/s41420-025-02293-3 (PMC11762296; doi:10.1038/s41420-025-02293-3)

Figure 2 C


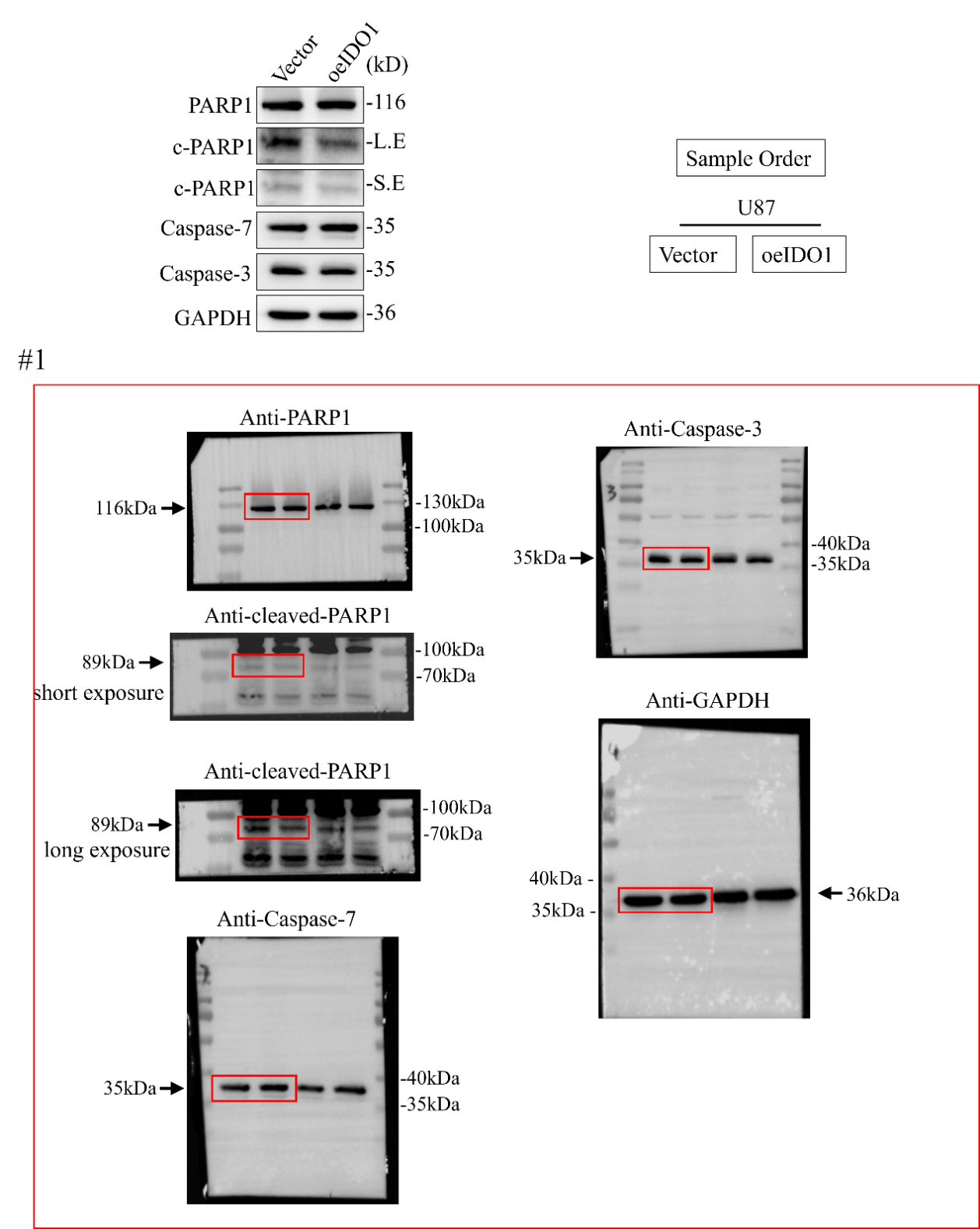


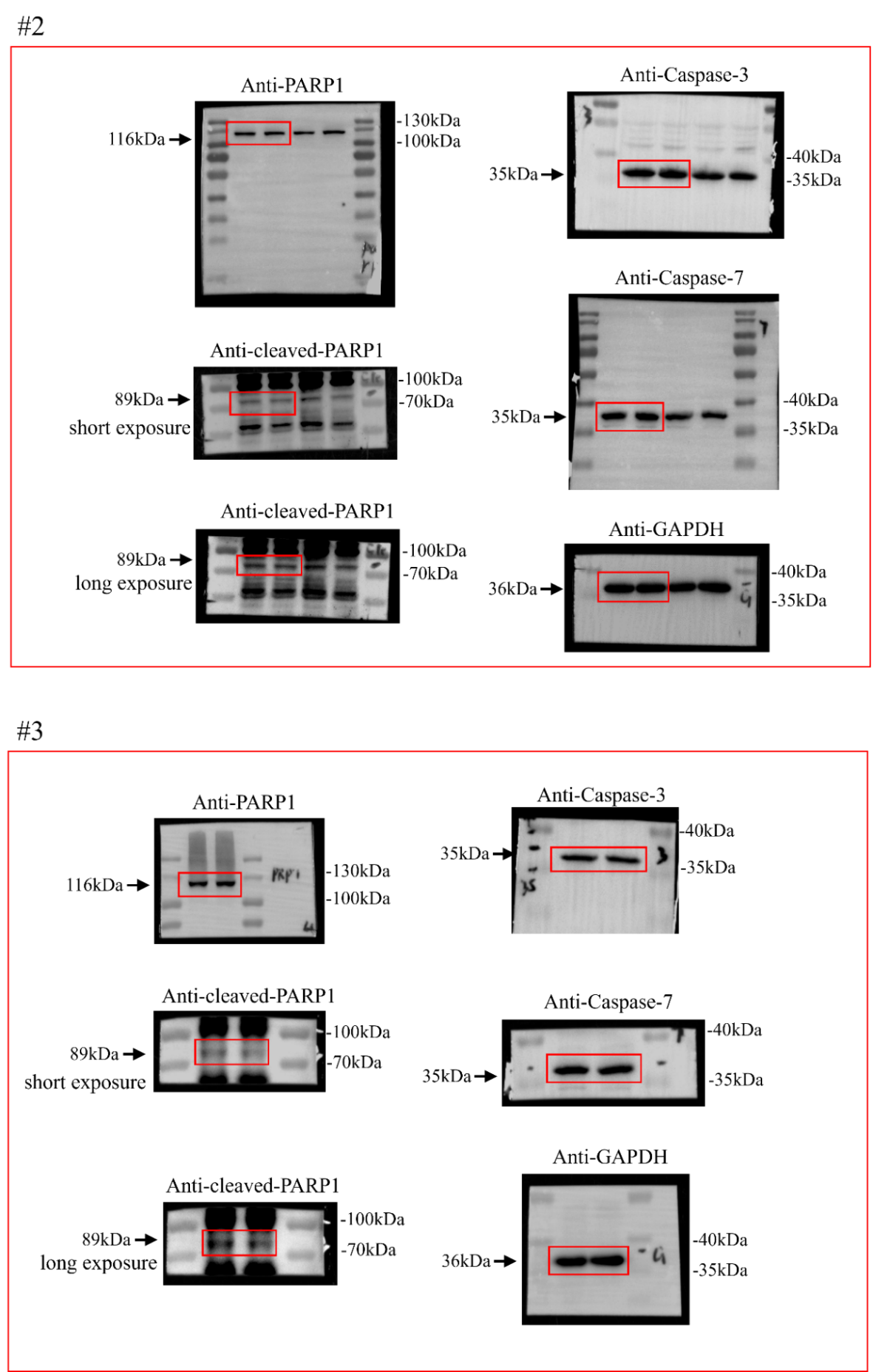


Figure 3 D


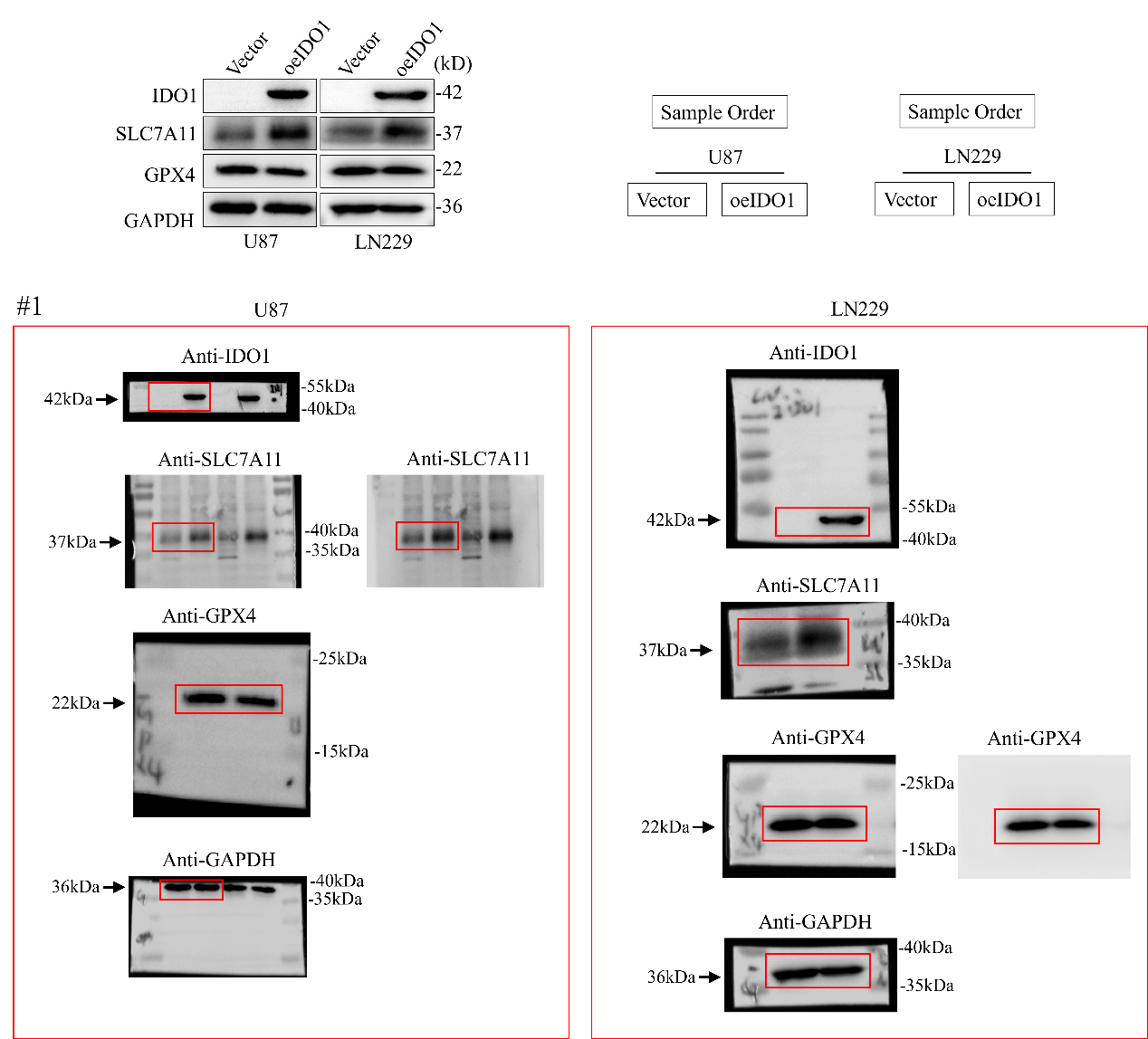


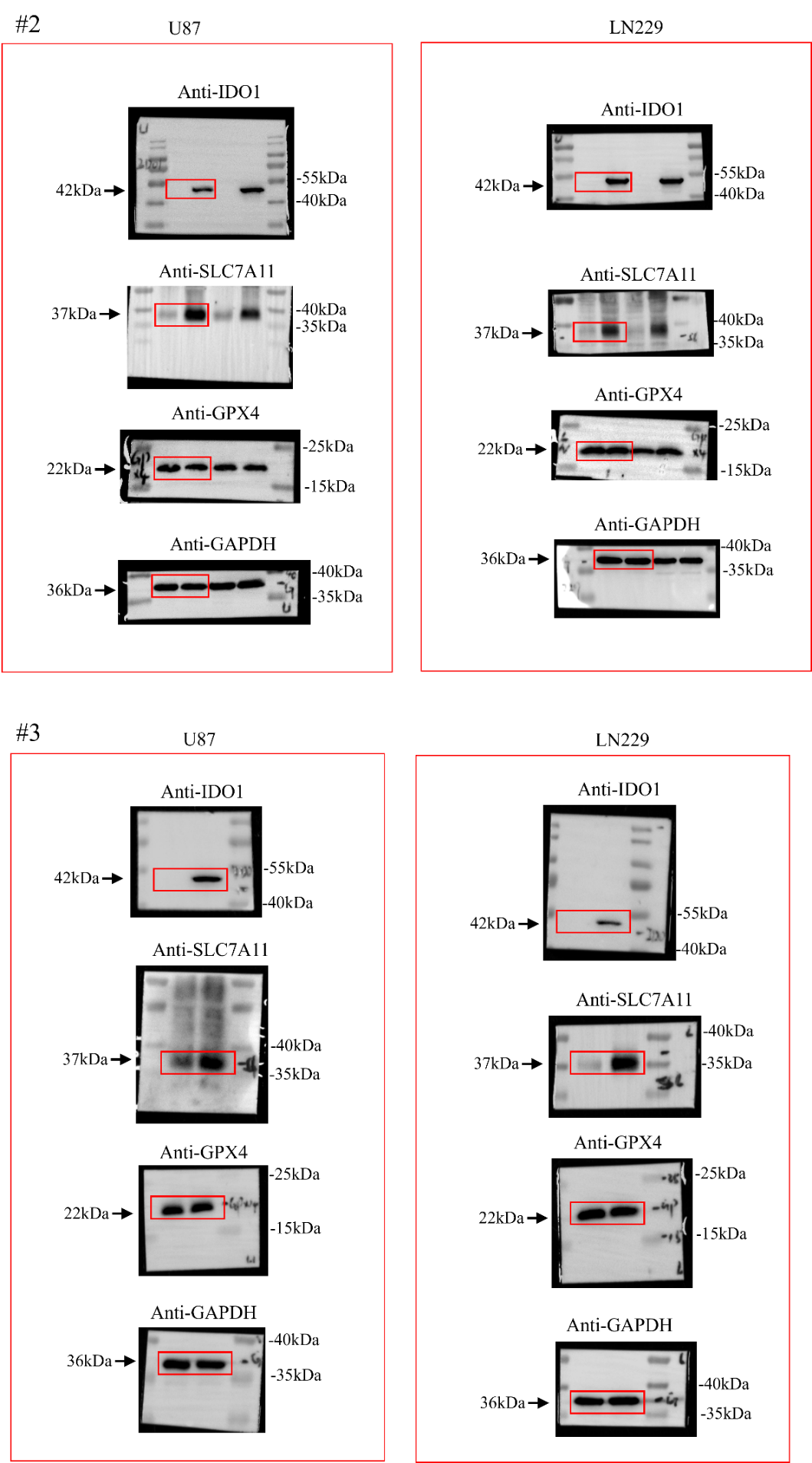


Figure 4 E


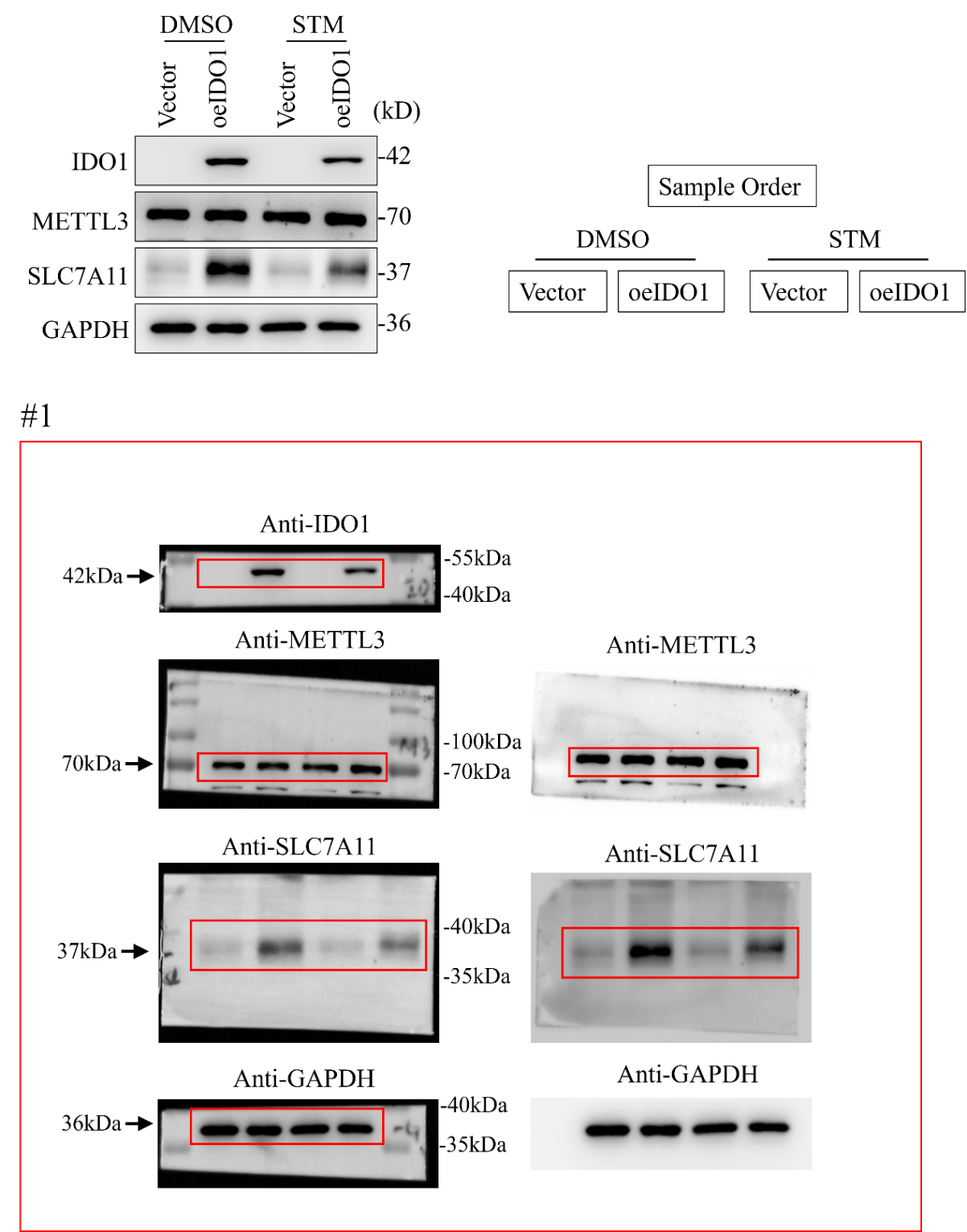


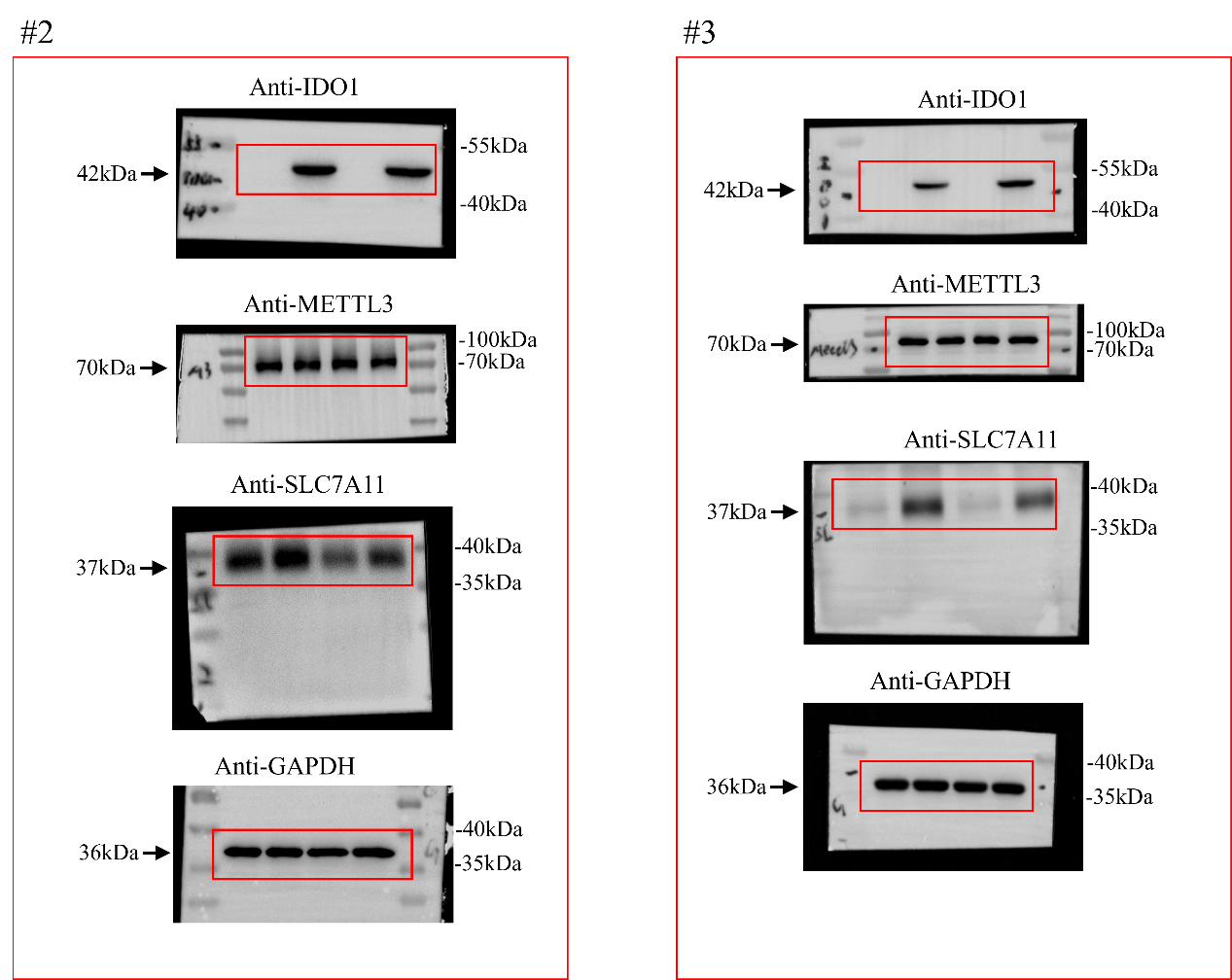


Figure 4 F


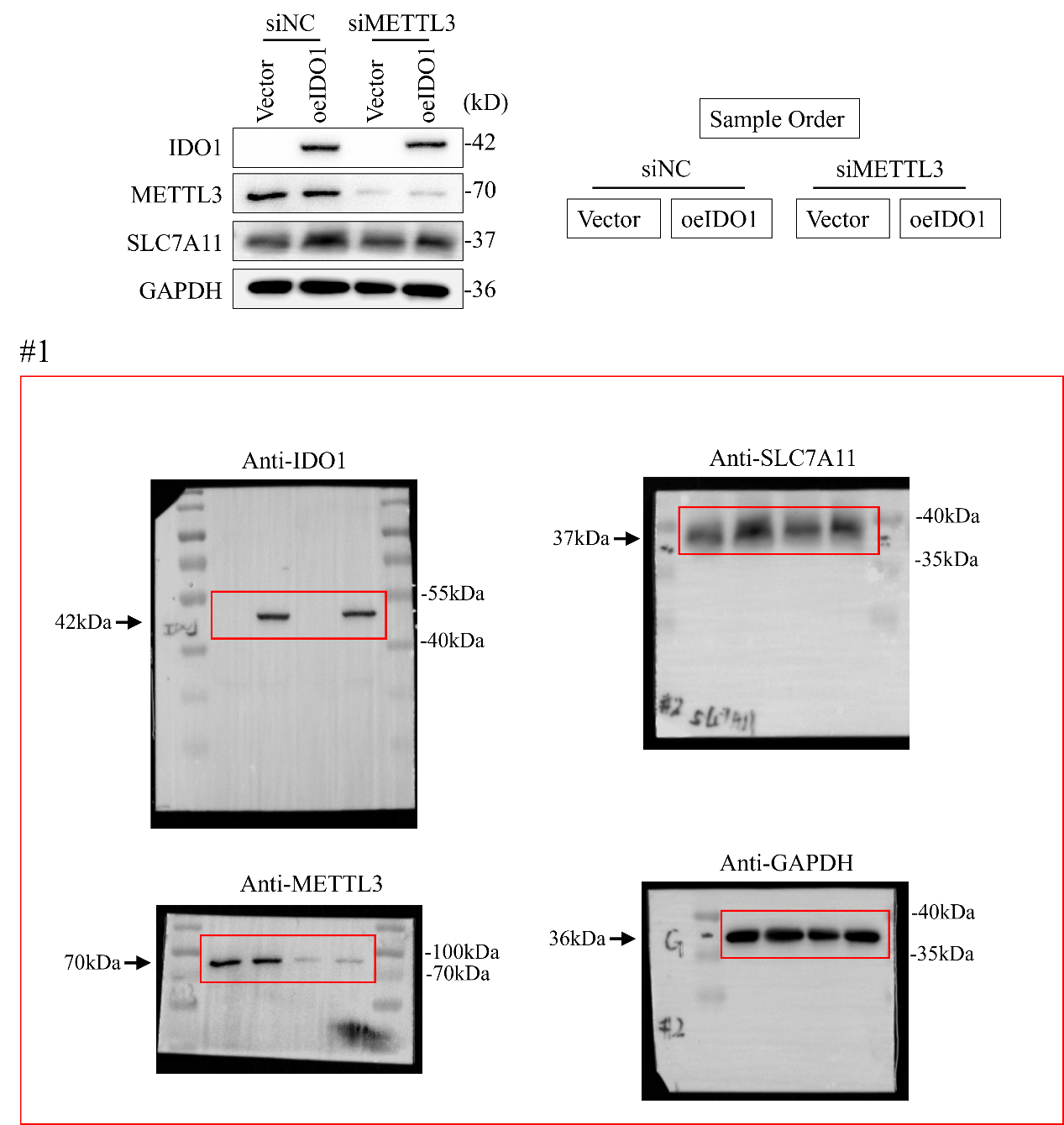


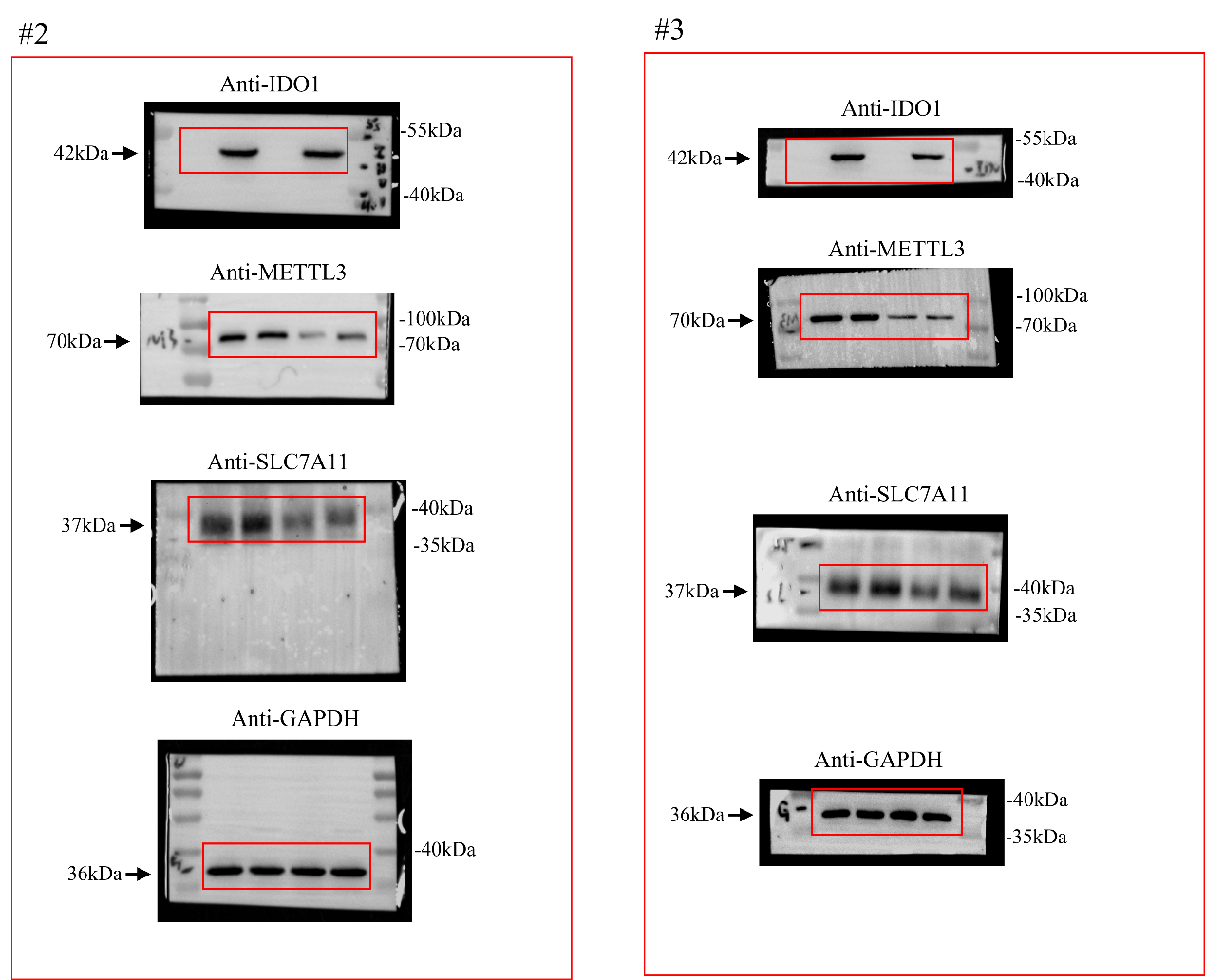


Figure 5 A


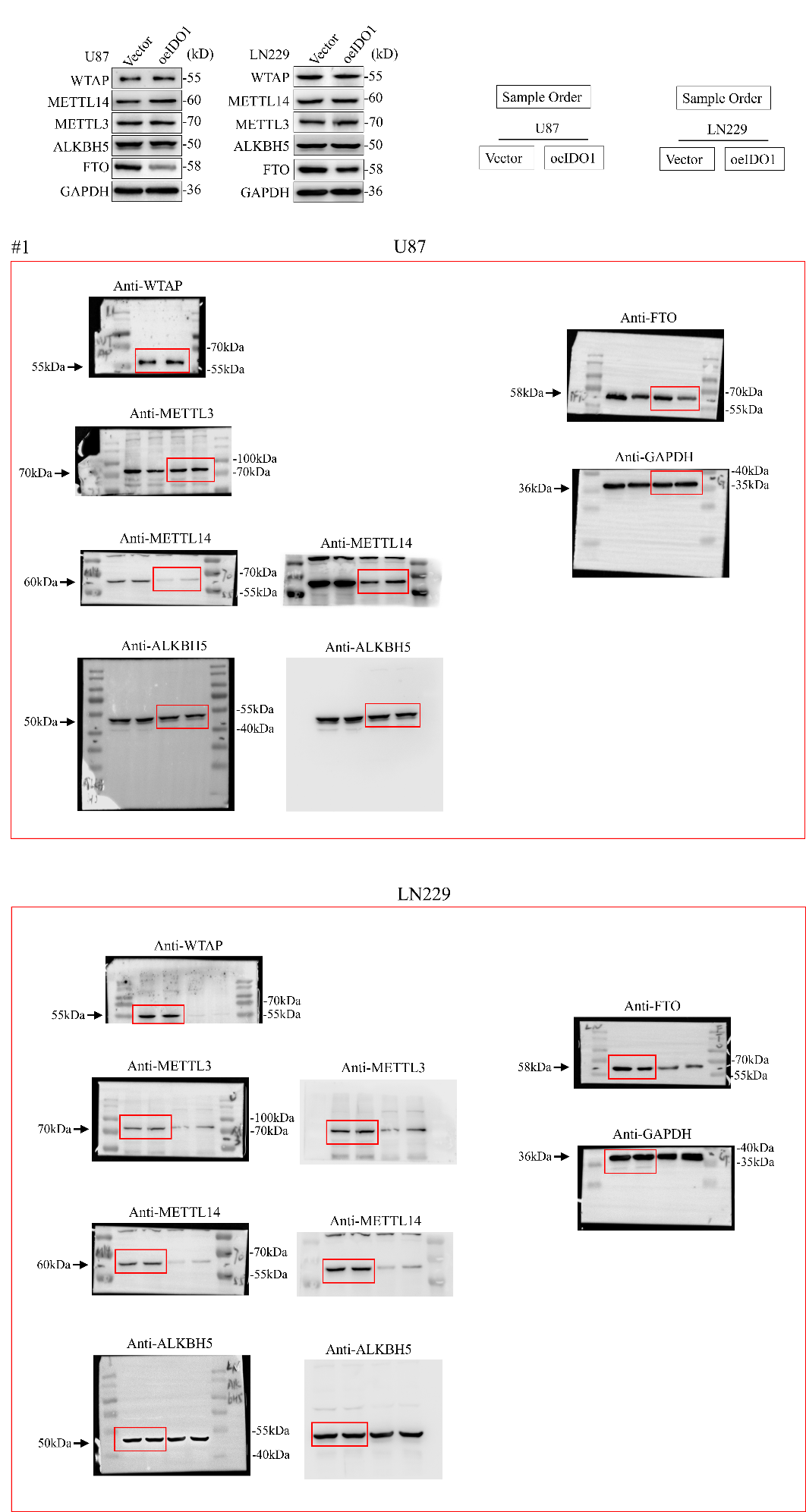


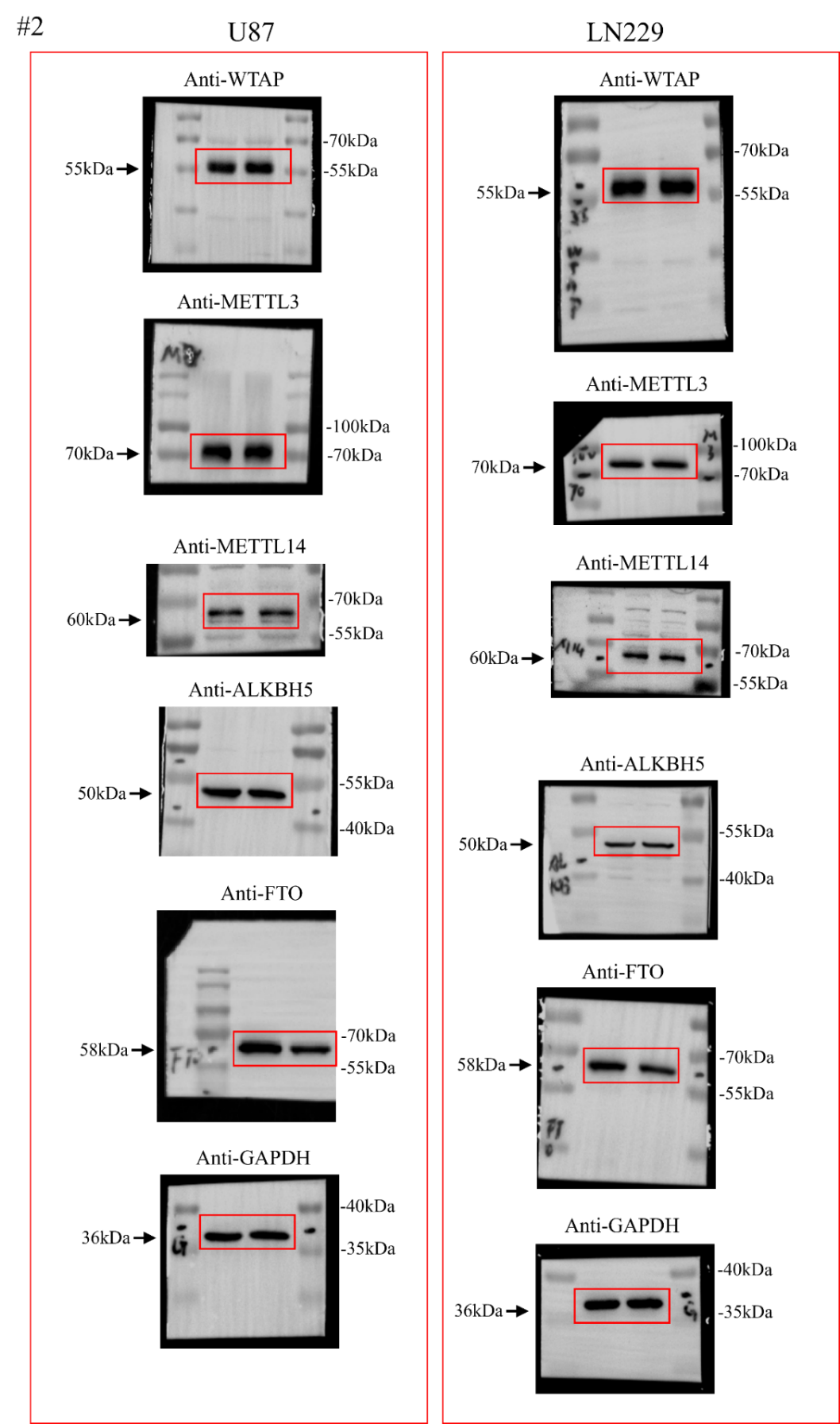


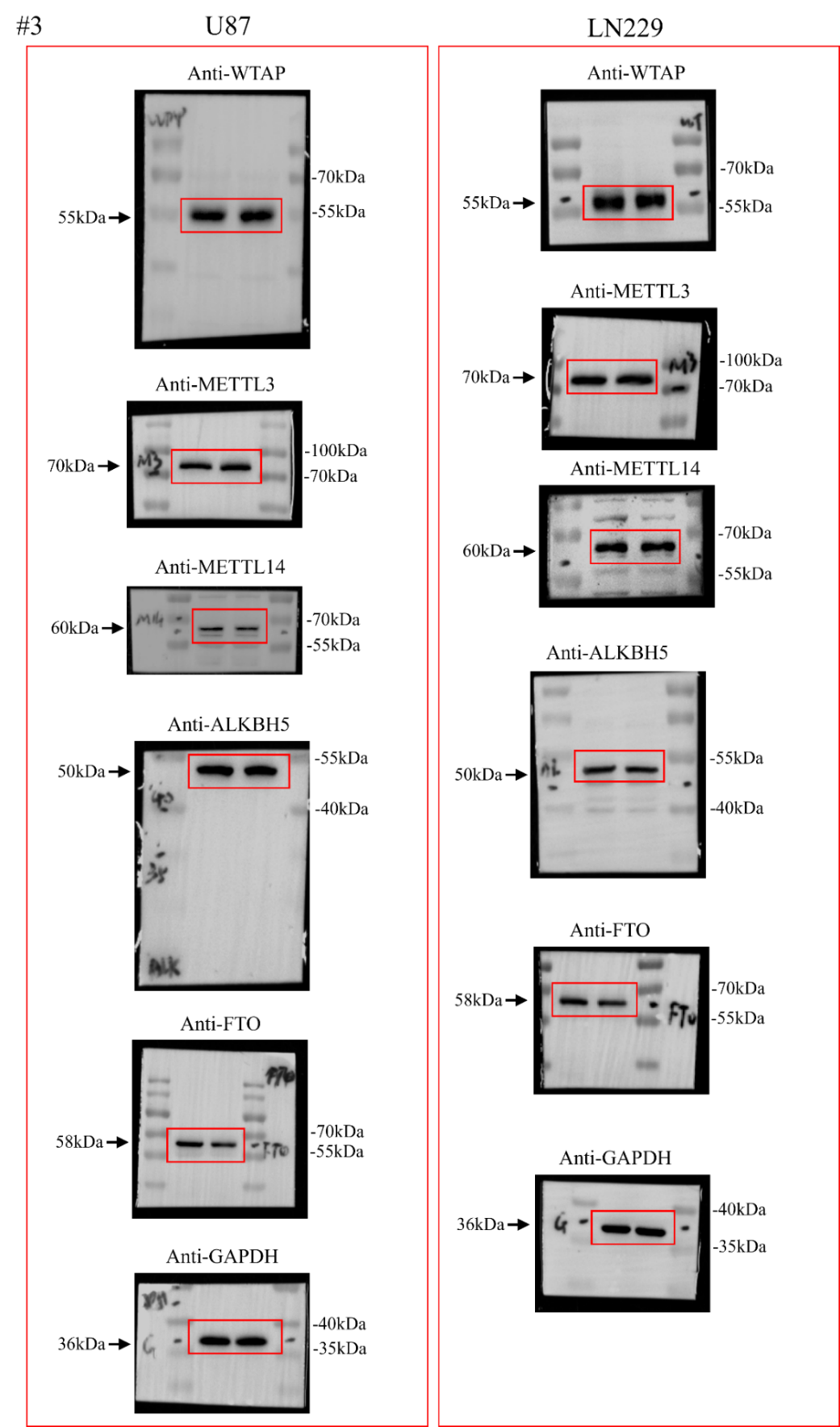


Figure 5 B


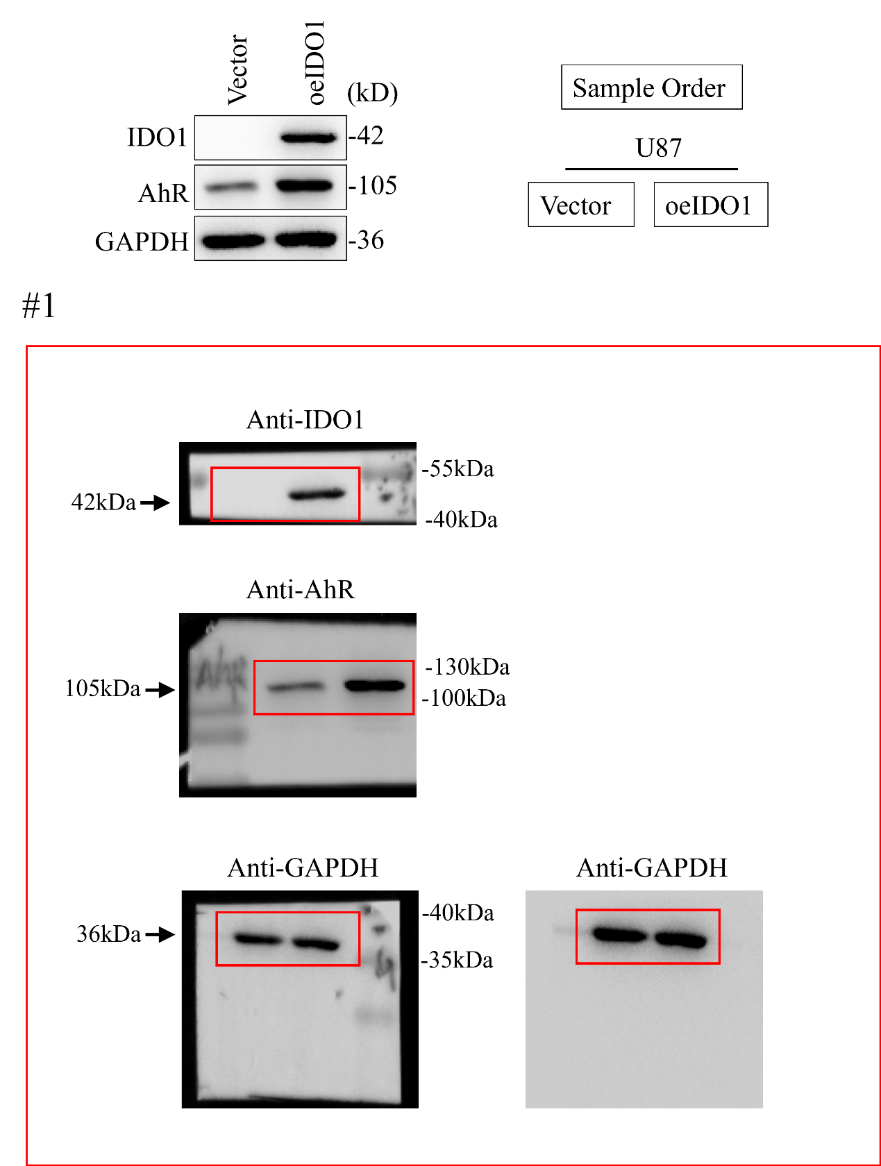


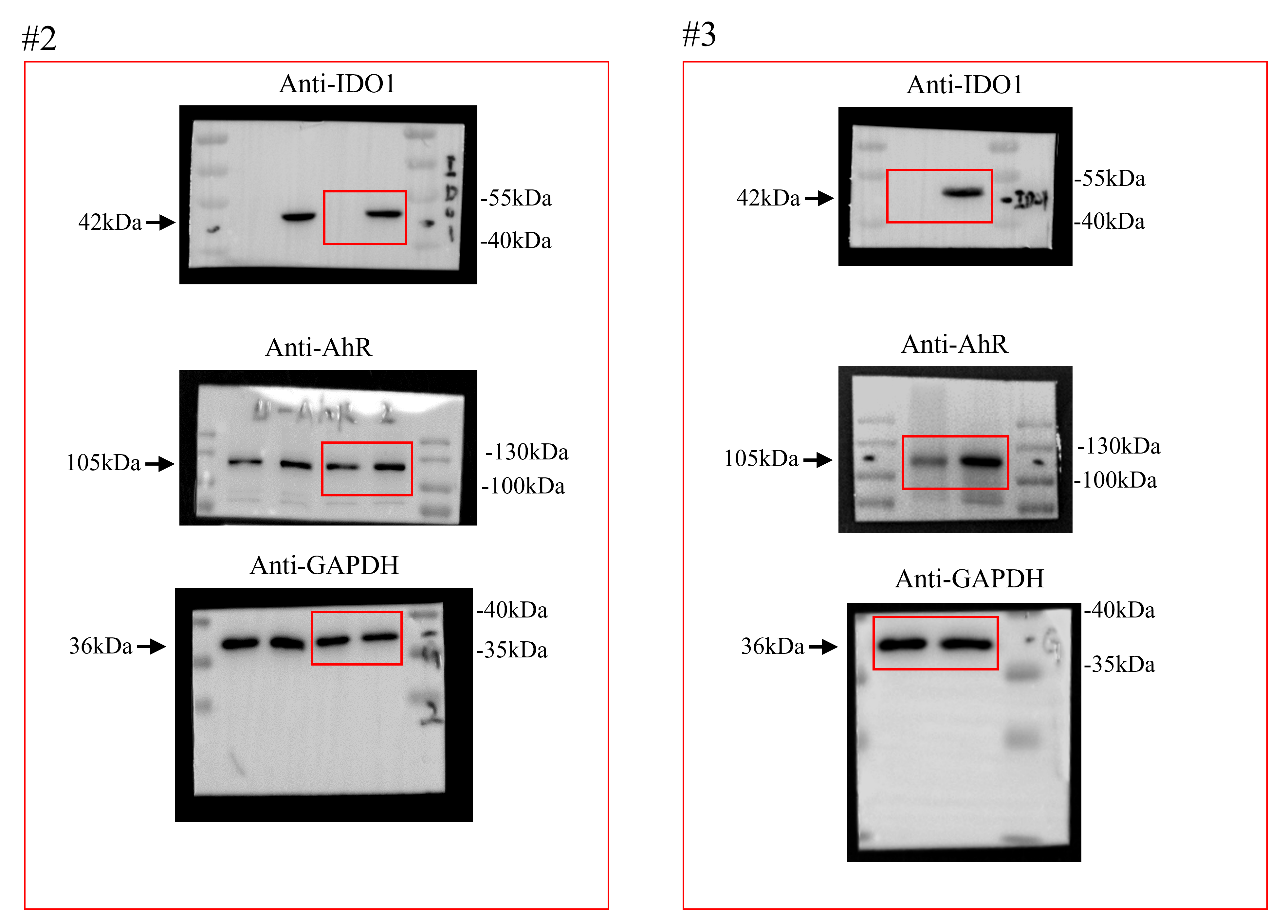


Figure 5 D


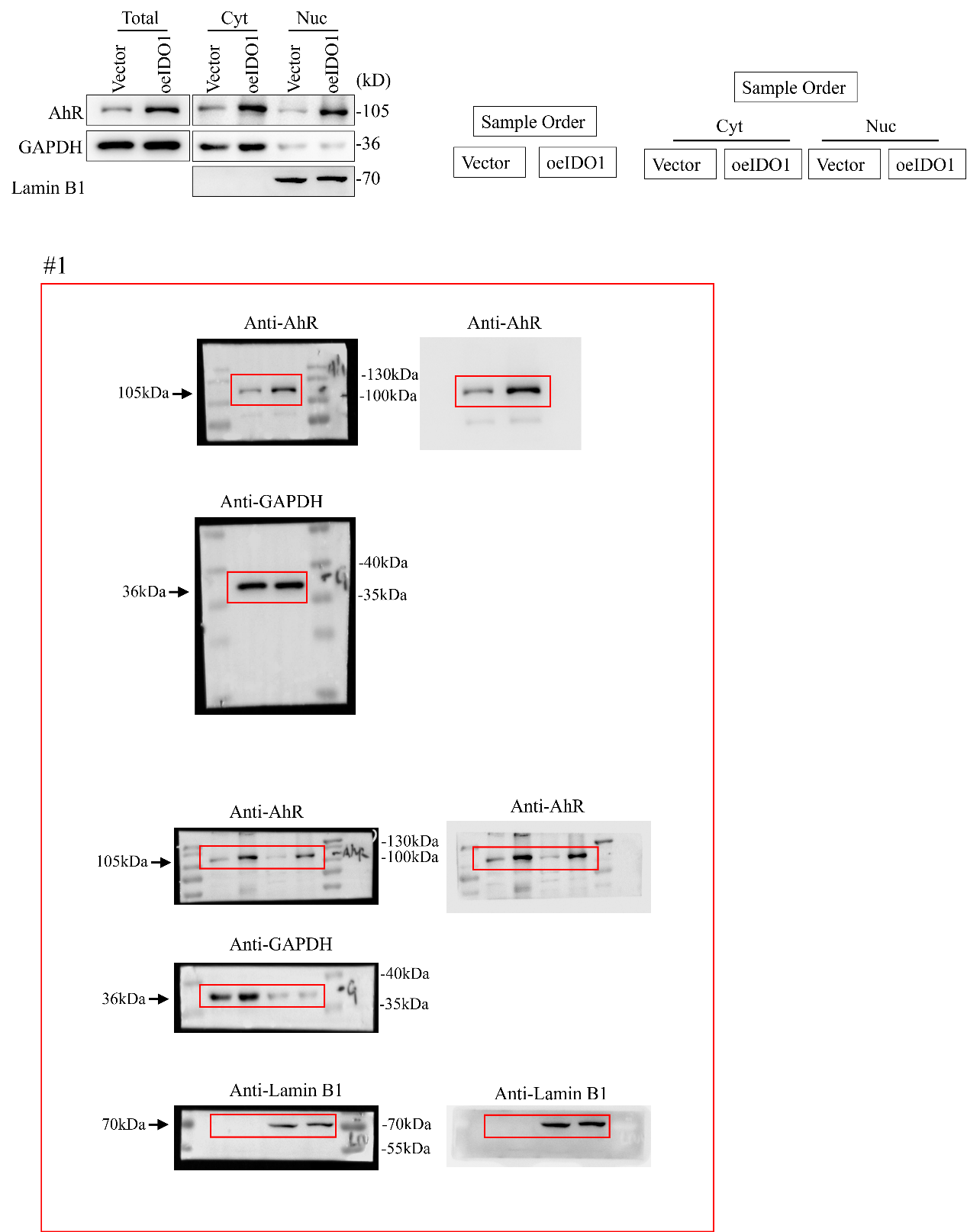


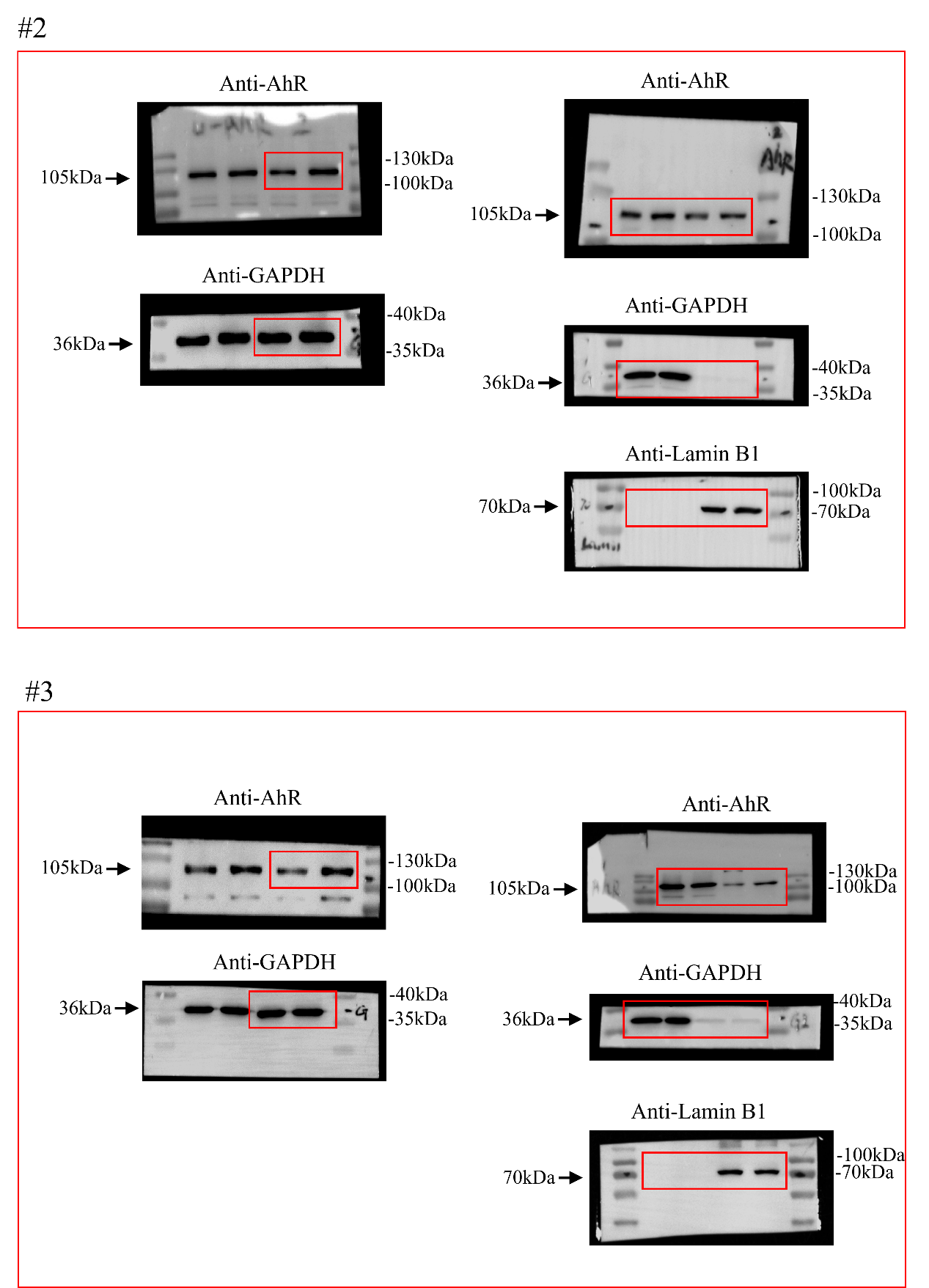


Figure 5 H


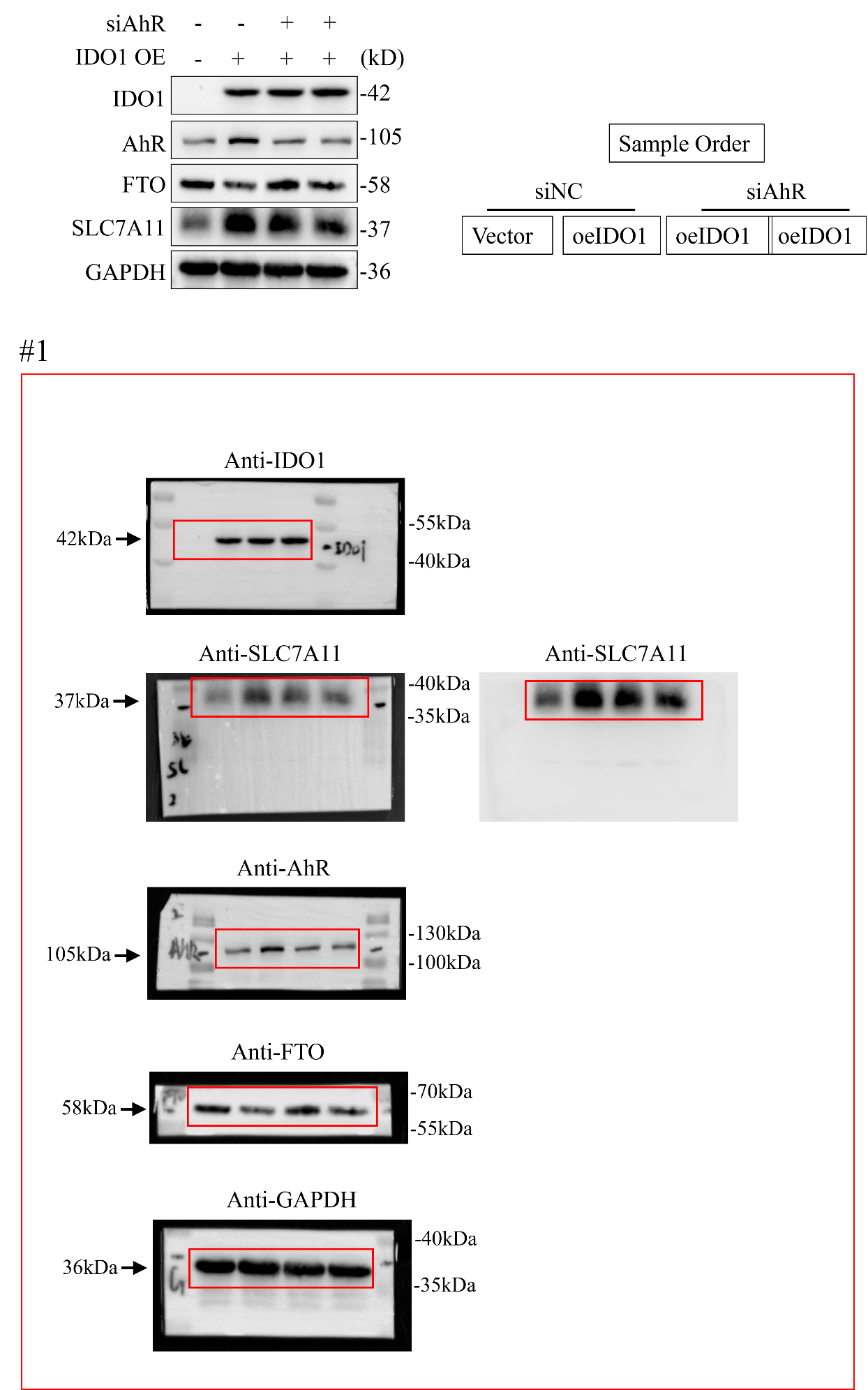


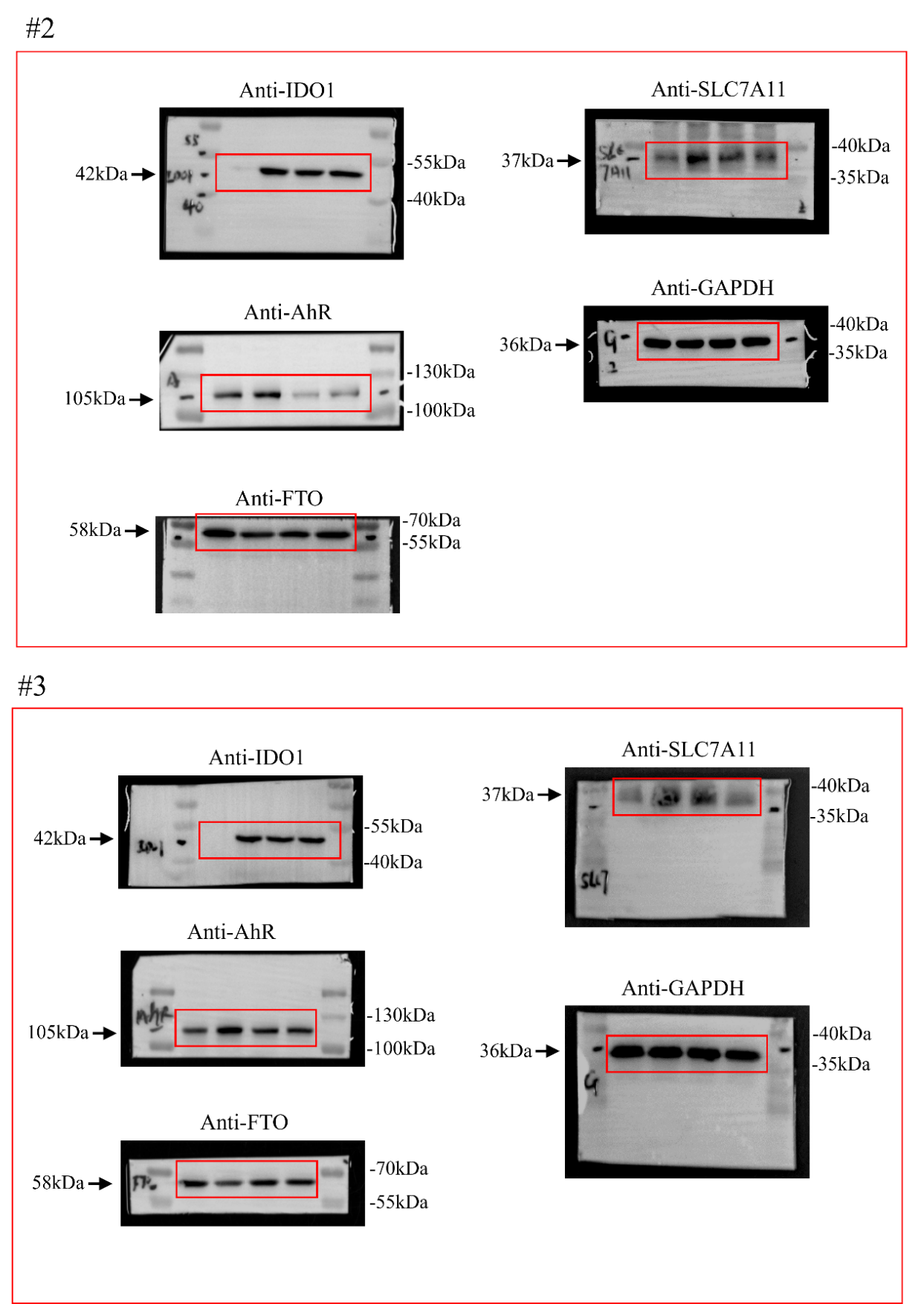


Figure 5 I


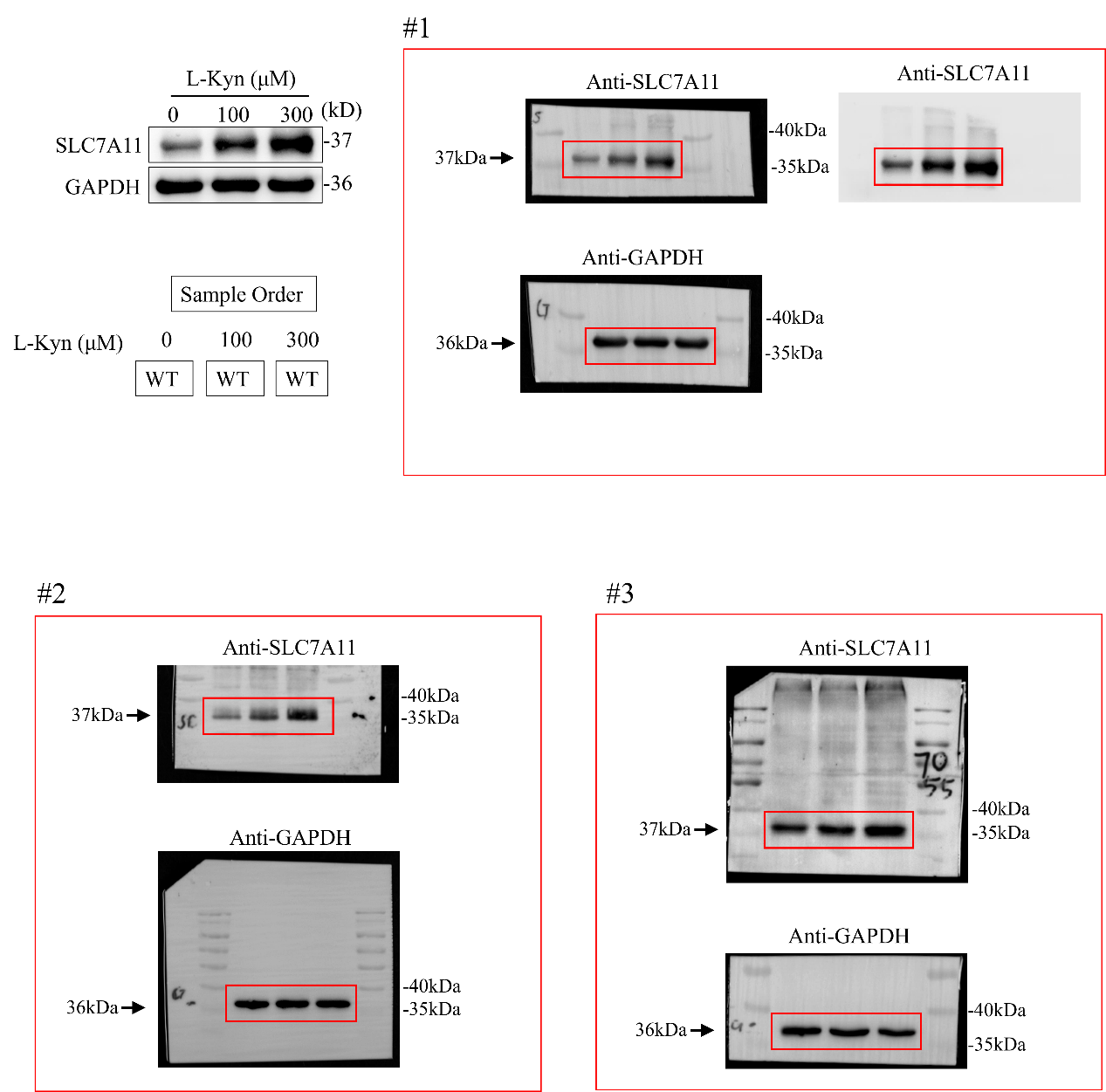


Figure S1 B


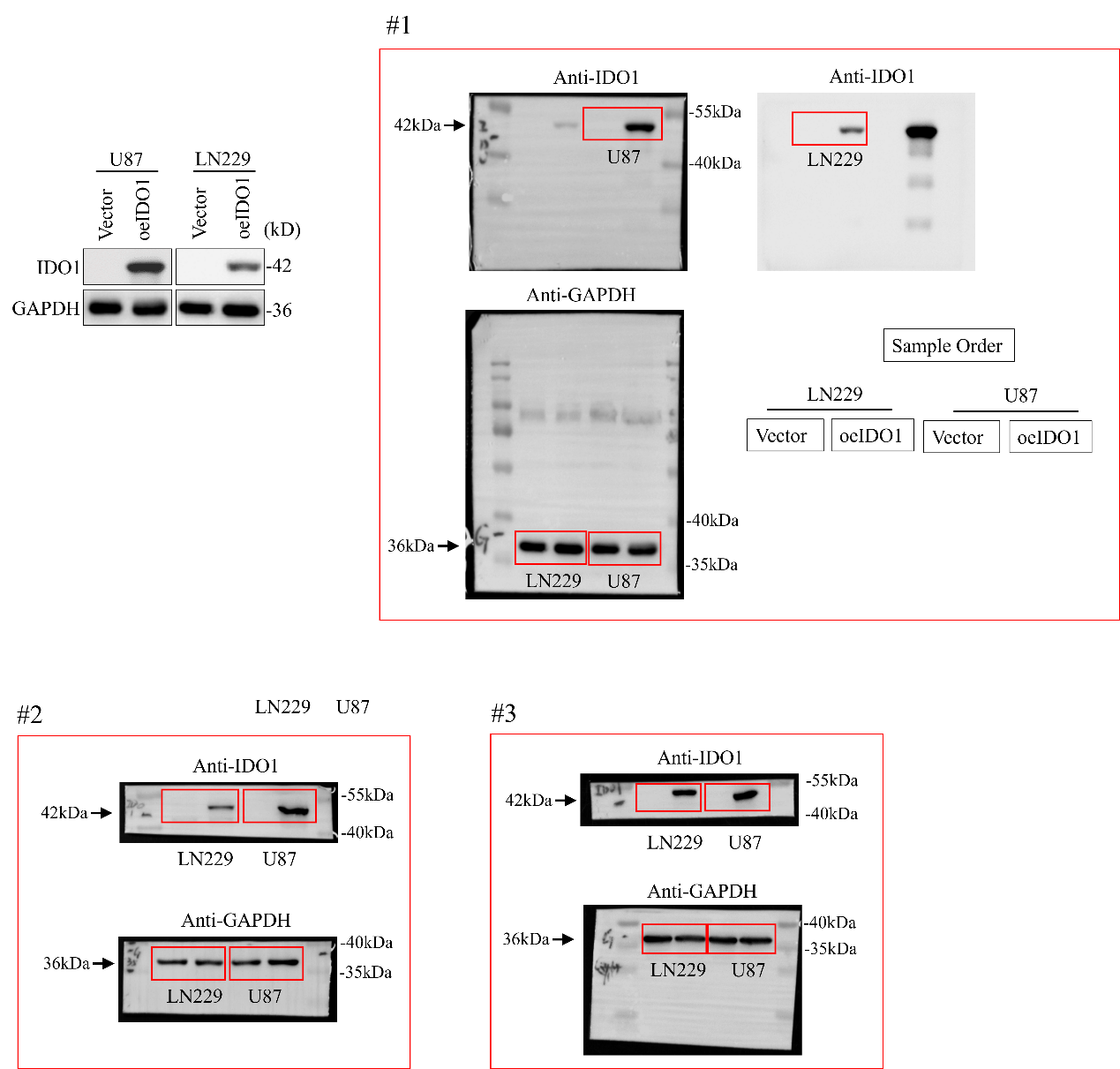

Supplement: Supplementary file 1 — Supplementary Information-Original Data [file 41420_2025_2293_MOESM1_ESM.docx]
