# Supplementary material for: IDO1 inhibits ferroptosis by regulating FTO-mediated m6A methylation and SLC7A11 mRNA stability during glioblastoma progression
Source: Cell Death Discov. 2025 Jan 25;11:22. doi: 10.1038/s41420-025-02293-3 (PMC11762296; doi:10.1038/s41420-025-02293-3)
Supplement: Supplementary file 2 — Supplementary Information-primers and antibody [file 41420_2025_2293_MOESM2_ESM.docx]

Supplementary Information

**Table S1. The primers and siRNA used in the study**

| **RT-qPCR** | **Forward (5’-3’)** | **Reverse (5’-3’)** |
| --- | --- | --- |
| H-GAPDH | CCATGGAGAAGGCTGGGG | CAAAGTTGTCATGGATGACC |
| H-IDO1 | TCTCATTTCGTGATGGAGACTGC | GTGTCCCGTTCTTGCATTTGC |
| H-SLC7A11 | ATGCAGTGGCAGTGACCTTT | CATGGAGCCAAAGCAGGAGA |
| H-GPX4 | GAGGCAAGACCGAAGTAAACTAC | CCGAACTGGTTACACGGGAA |
| **ChIP-qPCR** | **Forward (5’-3’)** | **Reverse (5’-3’)** |
| H-FTO-PP1 | GAACTACATGTGCGTGCCAC | GGTTCCCACCCTGTACCAG |
| H-FTO-PP2 | GAATGATTGGCTGTGCGTGG | CCAGGCTGGTCTCGAACTC |
| H-FTO-PP3 | CTGGACGTGGGGATAAAGGG | GAGTTGGCCCTGGAGTTCTG |
| H-FTO-PP4 | CGTTGCTATAGCGCCGACAG | ACAGGCTAGAGTCCAAGGAGA |
| **MeRIP-qPCR** | **Forward (5’-3’)** | **Reverse (5’-3’)** |
| H-SLC7A11- 5'UTR | GTTCTGGGACCCCGCTTTAC | GCAGGTTTATTGTGTGCGGG |
| H-SLC7A11- 3'UTR | TGGACTTGAGATCTTGGCAATCT | AACTGACTCCTTTTGTTTATCACC |
| siRNA | **Sequence** |  |
| H-si-SLC7A11 | CCAGAUAUGCAUCGUCCUUTT |  |
| H-si-AhR-1 | CCCACAAGAUGUUAUUAAUTT |  |
| H-si-AhR-2 | GUGACUUGUACAGCAUAAUTT |  |
| H-si-METTL3 | GCUCAACAUACCCGUACUATT |  |

**Table S2.** **Antibody information in the study**

| Antibody | Vendor | Catalog | Dilution (WB) |
| --- | --- | --- | --- |
| IDO1 | Cell Signaling Technology | 86630 | 1:1000 |
| xCT/SLC7A11 | Cell Signaling Technology | 12691 | 1:1000 |
| PARP1 | Cell Signaling Technology | 9542 | 1:1000 |
| GPX4 | Cell Signaling Technology | 59735 | 1:1000 |
| Cleaved-PARP1 | ABclonal technology | A22535 | 1:500 |
| METTL3 | Proteintech | 15073-1-AP | 1:1000 |
| FTO | Proteintech | 27226-1-AP | 1:1000 |
| ALKBH5 | Proteintech | 16837-1-AP | 1:3000 |
| METTL14 | Proteintech | 26158-1-AP | 1:1000 |
| WTAP | Proteintech | 60188-1-Ig | 1:1000 |
| AhR | Proteintech | 28727-1-AP | 1:1000 |
| Caspase 3 | Proteintech | 19677-1-AP | 1:1000 |
| Caspase 7 | Proteintech | 27155-1-AP | 1:1000 |
| GAPDH | Proteintech | 10494-1-AP | 1:20000 |
| m6A | Synaptic Systems | 202003 | 1:1000 |


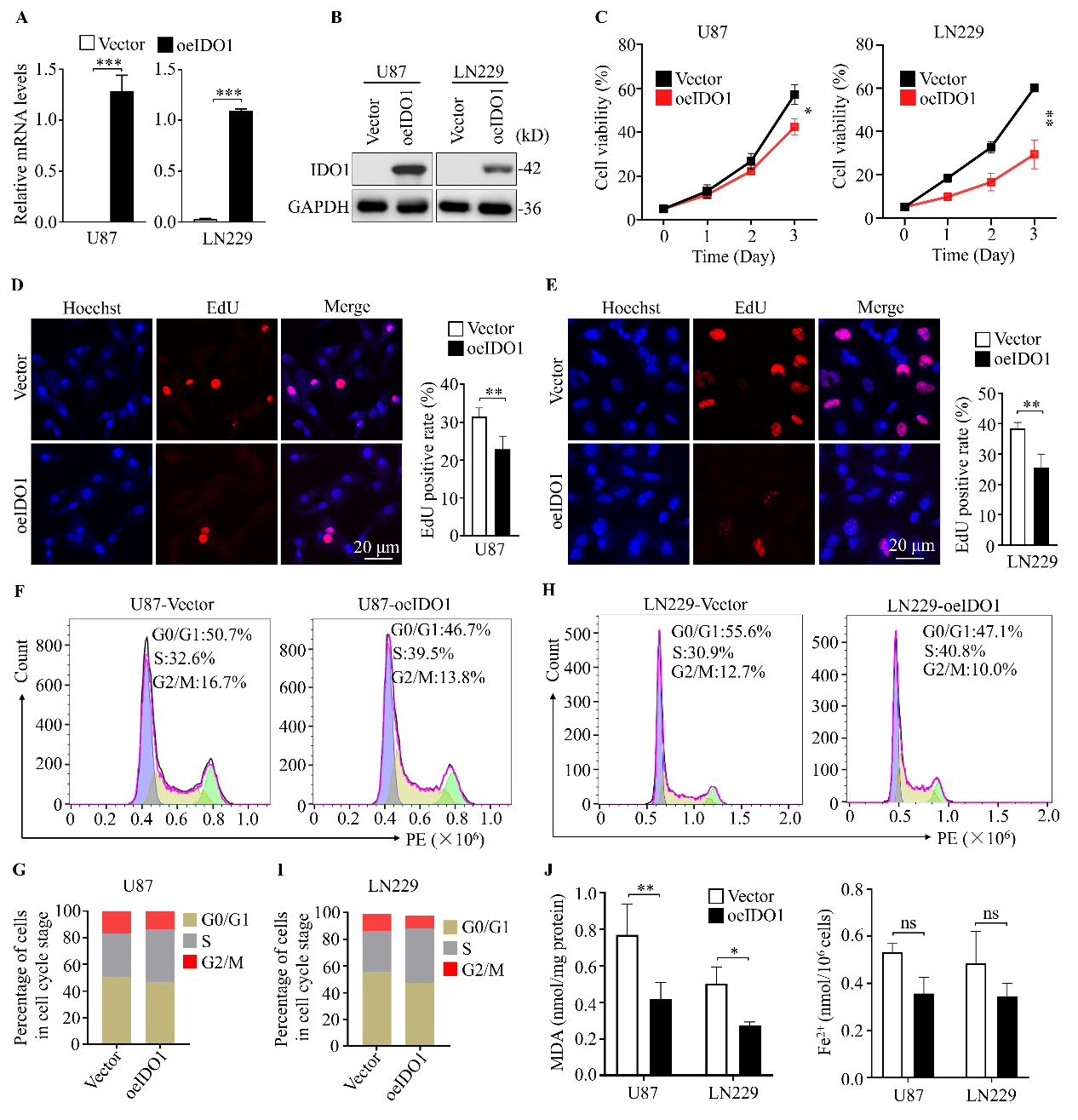


**Fig. S1 IDO1 suppressed lipid peroxidation generation and ferroptosis in GBM cells.**

**A** RT-qPCR was used to detect the IDO1 mRNA levels in U87 and LN229 cells with IDO1 overexpression (n = 3 biologically replicates). **B** Western blotting was used to detect the IDO1 protein expression in U87 and LN229 cells with IDO1 overexpression (n = 3 biologically replicates). **C** CCK-8 assay was carried out to determine GBM cells proliferation with IDO1 overexpression. **D, E** EdU assay was used to detect the DNA synthesis ability of GBM cells with IDO1 overexpression. **F, H** Cell cycle assay was performed by flow cytometry in the U87 cells **(F)** and LN229 cells **(H)** with IDO1 overexpression. **G, I** Cell cycle distribution analysis was carried out using FlowJo software in the U87 cells **(G)** and LN229 cells **(I)** with IDO1 overexpression. **J** The MDA and Fe2+ levels were detected in U87 and LN229 cells with IDO1 overexpression. The data are represented as the mean ± standard deviation (SD); *p <0.05, **p < 0.01, ***p <0.001, ns, not significant.


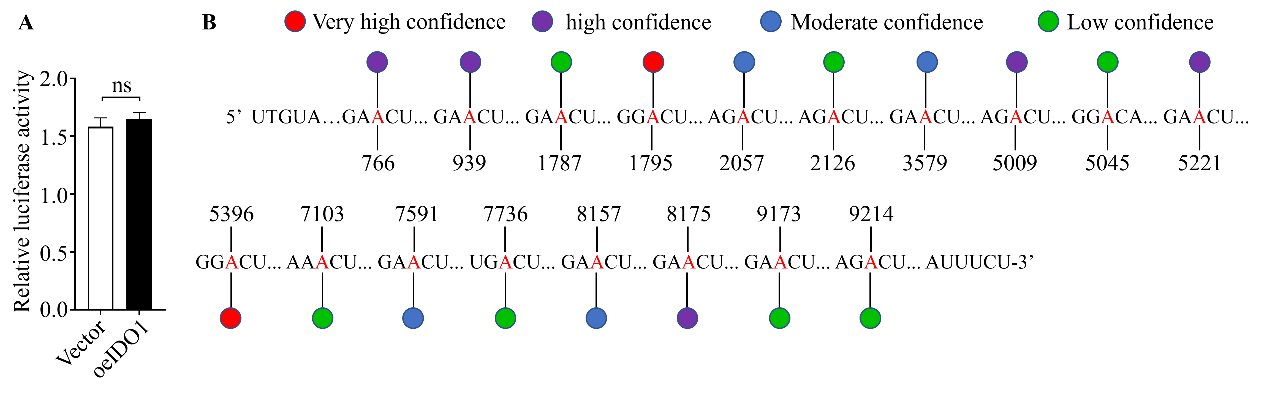
 **Fig. S2 IDO1 increased the SLC7A11 mRNA stability by increasing m6A deposition.**

**A** Luciferase reporter assays were used to determine the SLC7A11 promoter activity in the U87 cells with IDO1 overexpression. **B** Schematic diagram of predicted m6A modification sites in the SLC7A1 mRNA, data was obtained using SRAMP predicter. The data are represented as the mean ± standard deviation (SD); *p <0.05, **p < 0.01, ***p <0.001, ns, not significant.
